# A portable, low-cost, and open-source electrical impedance tomography system with a modular analog front-end based on STM32 and AD5933

**DOI:** 10.1016/j.ohx.2026.e00836

**Published:** 2026-09-11

**Authors:** Jerry Febrico, Basari

**Affiliations:** a Biomedical Engineering, Department of Electrical Engineering, Faculty of Engineering, Universitas Indonesia, Depok 16424, Indonesia; b Research Center for Biomedical Engineering, Universitas Indonesia, Depok 16424, Indonesia

**Keywords:** Electrical impedance tomography, Low-cost, Open-source, AD5933 impedance converter, Portable

## Abstract

Electrical impedance tomography (EIT) is a non-invasive, radiation-free medical imaging technique, but commercial devices remain costly and complex. This paper presents a portable, low-cost (95.3 USD) open-source 16-electrode time-difference EIT system based on the AD5933 impedance converter and an STM32F407VGT6 microcontroller. Its highly modular analog front-end enables switching between Load-in-the-Loop and Mirrored Howland Voltage-Controlled Current Source (VCCS) topologies, alongside AD620 module or INA118P instrumentation amplifiers. Utilizing an adjacent pattern, the computationally efficient system acquires 208 impedance data points in 2 s, which is highly suitable for static phantom imaging. Hardware validation at 50.68 kHz demonstrated exceptional measurement precision, yielding standard deviations between 0.54 and 6.33 , and a peak Signal-to-Noise Ratio (SNR) of 43.7 dB. Phantom experiments using a 3D-printed container filled with saline (966 mS/m) successfully evaluated the configurations using both conductive (stainless steel) and non-conductive (plastic) target anomalies across various positions. The 2D boundary voltages were reconstructed using the Back-Projection algorithm in the pyEIT library, achieving a maximum Structural Similarity Index Measure (SSIM) of 0.9242 and a minimum Root Mean Square Error (RMSE) of 28.29. This highly replicable, fully open-source platform offers a robust and accessible foundation for EIT research and education.

## Highlights

• This paper presents a portable, open-source, and low-cost (<$100) 16-electrode EIT hardware driven by an AD5933 impedance converter and STM32F407VGT6 microcontroller.
• The system features a versatile analog front-end, allowing users to conveniently select between different VCCS topologies (Load-in-the-Loop or Mirrored Howland) and instrumentation amplifiers (AD620 or INA118P).
• Leveraging the STM32′s computational efficiency, the device rapidly acquires a full frame of 208 boundary voltage measurements in just 2 s, demonstrating good signal stability (SNR up to 43.7 dB) and good repeatability (ranging from 0.54 Ω to 6.33 Ω).
• Validated on a custom 3D-printed saline phantom, the system successfully reconstructs high-quality 2D images (SSIM of 0.9242) using the Python-based pyEIT framework, providing an easily replicable platform for EIT research and education.

## Graphical abstract

**Figure:**
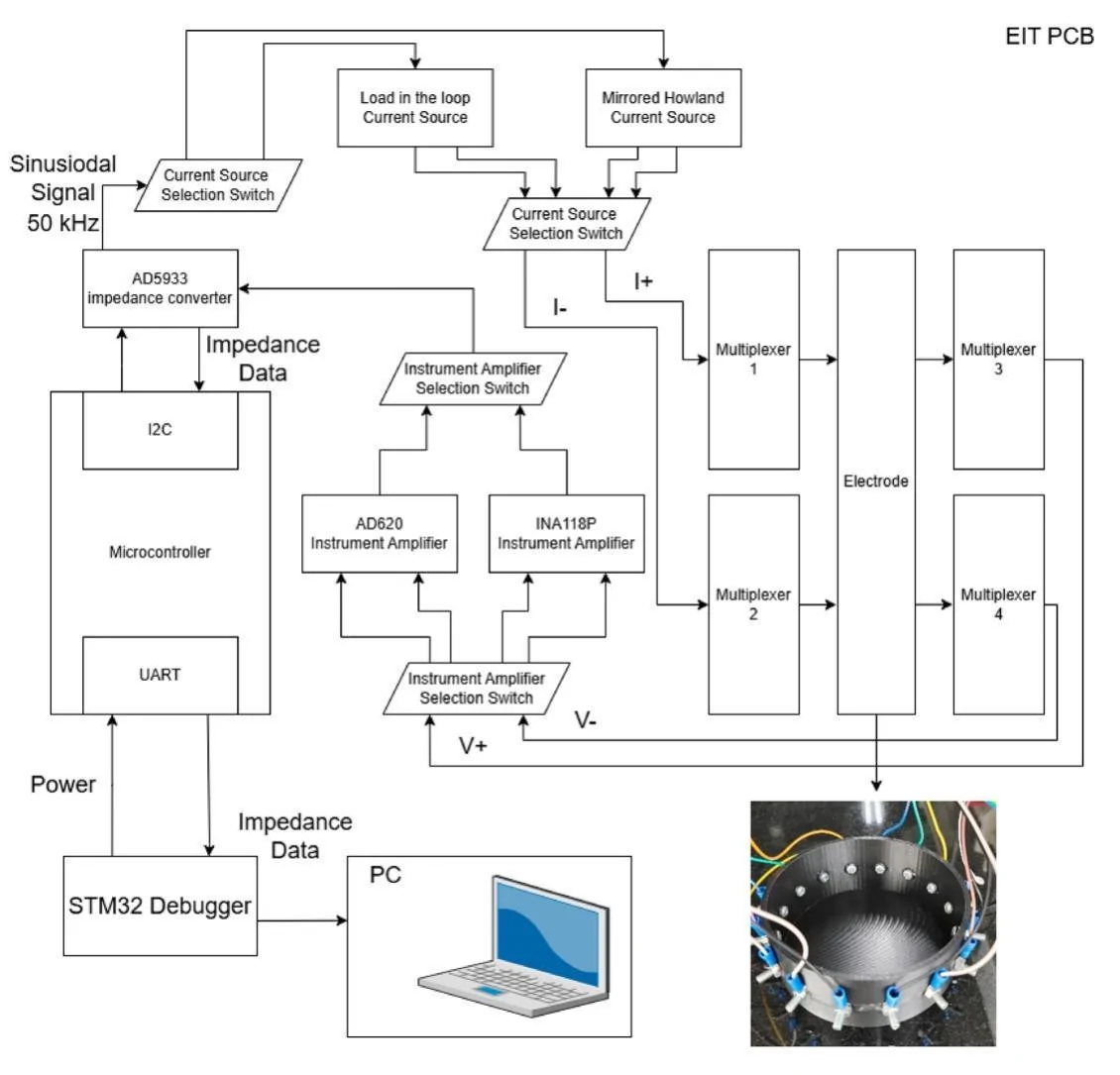

Specifications table

**Table:** 

Hardware name	STM32 based EIT System
Subject area	Medical Educational tools and open-source alternatives to existing infrastructure
Hardware type	Imaging tools
Closest commercial analog	No commercial analog available.
Open source license	CERN-OHL, MIT License and, CC BY 4.0
Cost of hardware	$ 95.3 USD
Source file repository	https://data.mendeley.com/datasets/9skj7cmwsf/2 or https://doi.org/10.17632/9skj7cmwsf.2

## 1 Hardware in context

In respiratory care, continuous bedside monitoring is critical for managing diseases such as pulmonary edema and Chronic Obstructive Pulmonary Disease (COPD), which remain significant global health burdens, particularly in developing nations. In this context, Electrical Impedance Tomography (EIT) holds significant potential to support respiratory management due to its non-invasive nature and suitability for repeated, real-time bedside monitoring without radiation risks [1]. EIT is a portable, non-invasive, and radiation-free imaging technique that estimates the internal conductivity distribution of the thorax by injecting small currents and measuring the resulting boundary voltages across the skin. Compared to other imaging techniques such as CT scans or magnetic resonance imaging (MRI), EIT offers advantages in lower operational costs [2]. Additionally, its non-ionizing imaging principle ensures safety for continuous patient observation [3]. While CT and MRI provide millimeter spatial resolution, they expose patients to ionizing radiation or require massive, non-portable infrastructure. Ultrasound is portable and radiation-free, but its image quality is heavily dependent on operator skill and offers poor contrast for air-filled lungs. EIT occupies a unique clinical niche by sacrificing spatial resolution in exchange for continuous, non-ionizing, real-time bedside functional monitoring [2].

As summarized in Table 1, several open-source and low-cost EIT systems have been developed recently to democratize access to medical imaging. For instance, Muñoz et al. [4] introduced a highly affordable 2D bioimpedance system utilizing the AD5933 impedance converter. However, while the system is less expensive, this design is limited to an 8-electrode configuration. Conversely, systems that support standard 16 to 32 electrode arrays often incur significantly higher hardware costs. The wearable EIT system introduced by Creegan et al. [5] and the Arduino Mega based EIT system by Zamora et al. [6] both exceed 250 USD in fabrication costs. Similarly, a recently proposed wide-bandwidth (1 kHz to 1 MHz) STM32-based platform by Hall et al. offers extensive modularity but comes at a higher cost of 330 USD [7]. Despite these significant advancements, highly modular systems tend to introduce greater hardware complexity and require an external power source. In addition to lowering hardware costs, recent studies highlight the importance of easy-to-use software. For instance, the MATLAB-based graphical user interface (GUI) provides a practical solution to streamline the EIT imaging process [8]. This research presented a low-cost, beginner-friendly for students and early-career researchers that exploring EIT. While advanced architectures like Field-Programmable Gate Arrays (FPGAs) offer superior processing performance, they demand significantly higher fabrication costs and a steep technical learning curve.

**Table 1:** State of the art.

Authors	Electrode Number	Frequency	Microcontroller	Image Reconstruction Platform	Cost
Yang et al. [3]	32	10 kHz – 20 kHz	Arduino Due	EIDORS	−
Muñoz et al. [4]	8	50 kHz	Arduino Mega	EIDORS	80.5 USD
Creegan et al. [5]	16–––32	10 kHz	Teensy 3.6	pyEIT	260 USD
Zamora et al. [6]	16	50 kHz	Arduino Mega	EIDORS	250 USD
Henry W. Hall et al. [7]	16–––32	1 kHz − 1 MHz	STM32G474VET6	pyEIT	330 USD
Mohamedali et al. [9]	16	50 kHz	Arduino Mega	EIDORS	63.61 USD
Deng Qilong et al. [10]	16	50 kHz	16 pcs STM32	−	−
Ain et al. [11]	16	4–8 kHz	Arduino Mega	GNU Octave	−
**This work**	**16**	**50 kHz**	**STM32F407VGT6**	**pyEIT**	**95.3 USD**

The hardware presented in this work introduces a portable and low-cost EIT system built upon the STM32 microcontroller. This design integrates the microcontroller alongside an analog front-end utilizing the AD5933 for 50 kHz signal generation, multiplexer, and the INA118P for precise instrumentation amplification into a custom PCB. By leveraging the computational efficiency of the STM32 architecture, the system rapidly executes a 16-electrode adjacent data acquisition cycle. The novelty of this system lies in its highly modular analog front-end and a seamless, end-to-end open-source software pipeline. To support the hardware, a Python-based graphical user interface (GUI) was developed to control and streamline the automated data acquisition process, which is integrated directly with pyEIT to solve the inverse problem. Consequently, this work provides a highly reproducible, low-cost, and robust foundation for researchers advancing toward EIT solutions. Please note that this prototype is strictly intended for educational and non-human phantom experiments, as it does not currently feature medical-grade isolation compliant with IEC 60601–1 standards.

## 2 Hardware description

**Fig. 1:**
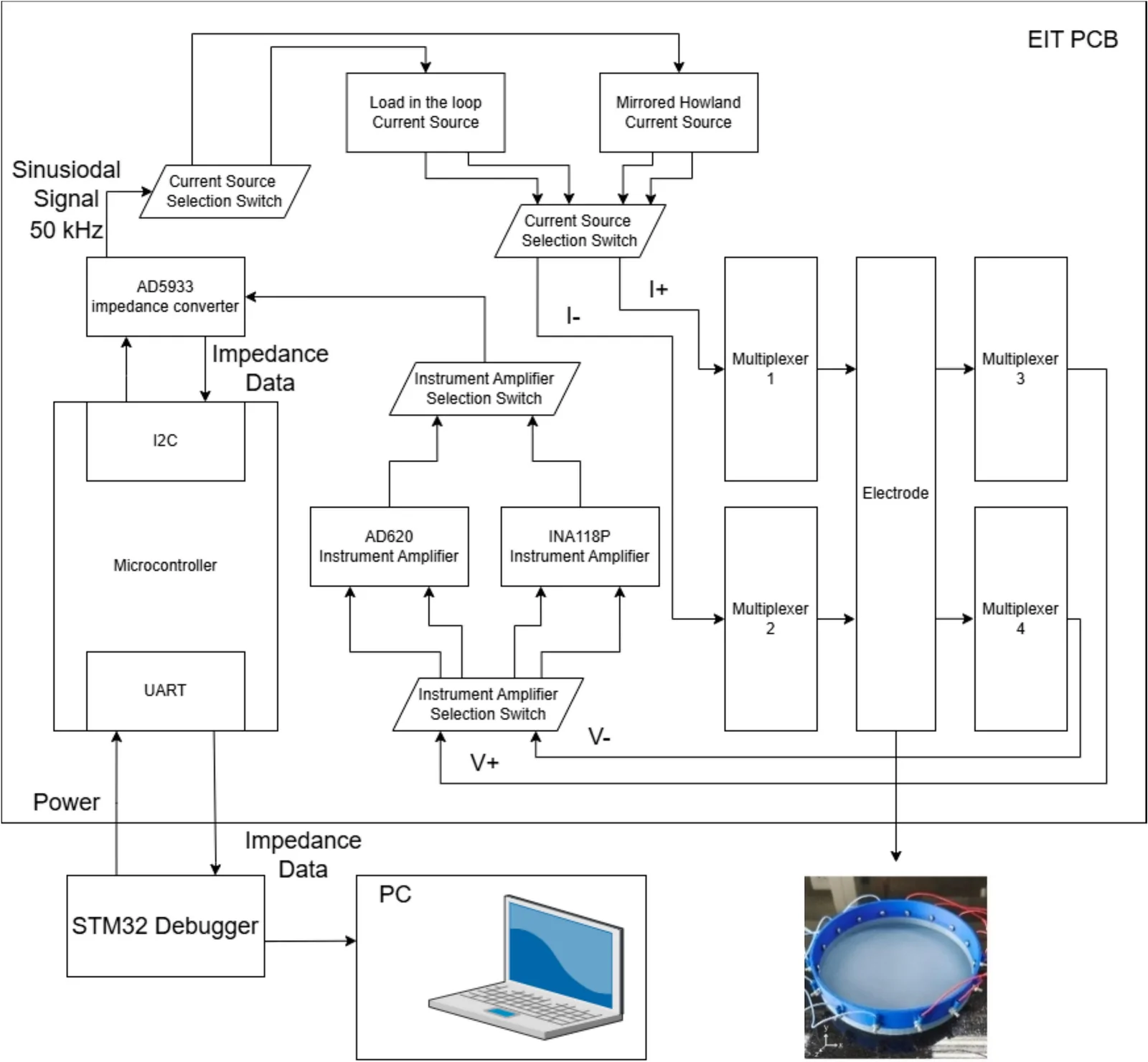
Diagram of the proposed EIT system architecture.

The proposed EIT system consists of three main sections: the electronics, the electrode container, and the calibration and visualization software, which is operated via a custom-built graphical user interface (GUI) [12]. Fig. 1 illustrates the diagram of the proposed EIT system architecture, detailing the hardware components, signal pathways, and control flow. The entire circuit comprising the microcontroller, impedance converter with a Direct Digital Synthesizer (AD5933), Voltage-Controlled Current Source (VCCS), multiplexers, and instrumentation amplifiers (IAs) is integrated into a single PCB measuring 177.8 x 155.83 mm. The STM32F407VGT6 microcontroller controls the AD5933 via I2C communication and manages the switching of the 16-channel multiplexer array. Furthermore, it processes the imaginary and real data from the AD5933 to calculate the impedance value and transmits this data via UART to a personal computer for image reconstruction.

Within this PCB configuration, two different types of VCCS and two types of instrumentation amplifiers (IAs) are provided. This design allows researchers to investigate and compare which circuit configuration yields better performance. Switching between the circuit paths is facilitated by manual switches installed directly on the PCB, with the routing numbers clearly printed on the board for user convenience. The two VCCS designs included are the Load-in-the-Loop Current Source and the Mirrored Howland Current Source. Similarly, the instrumentation amplifier options consist of an AD620 module and an INA118P chip. The PCB is assembled using IC sockets, female headers, and through-hole resistors to make it easier for users to modify the board, replace damaged components, and reduce overall manufacturing costs. All components required to build this system are clearly listed in the Bill of Materials (BOM), complete with purchase links from online marketplaces such as DigiKey and AliExpress, which offer worldwide shipping.

### 2.1 AD5933

**Fig. 2:**
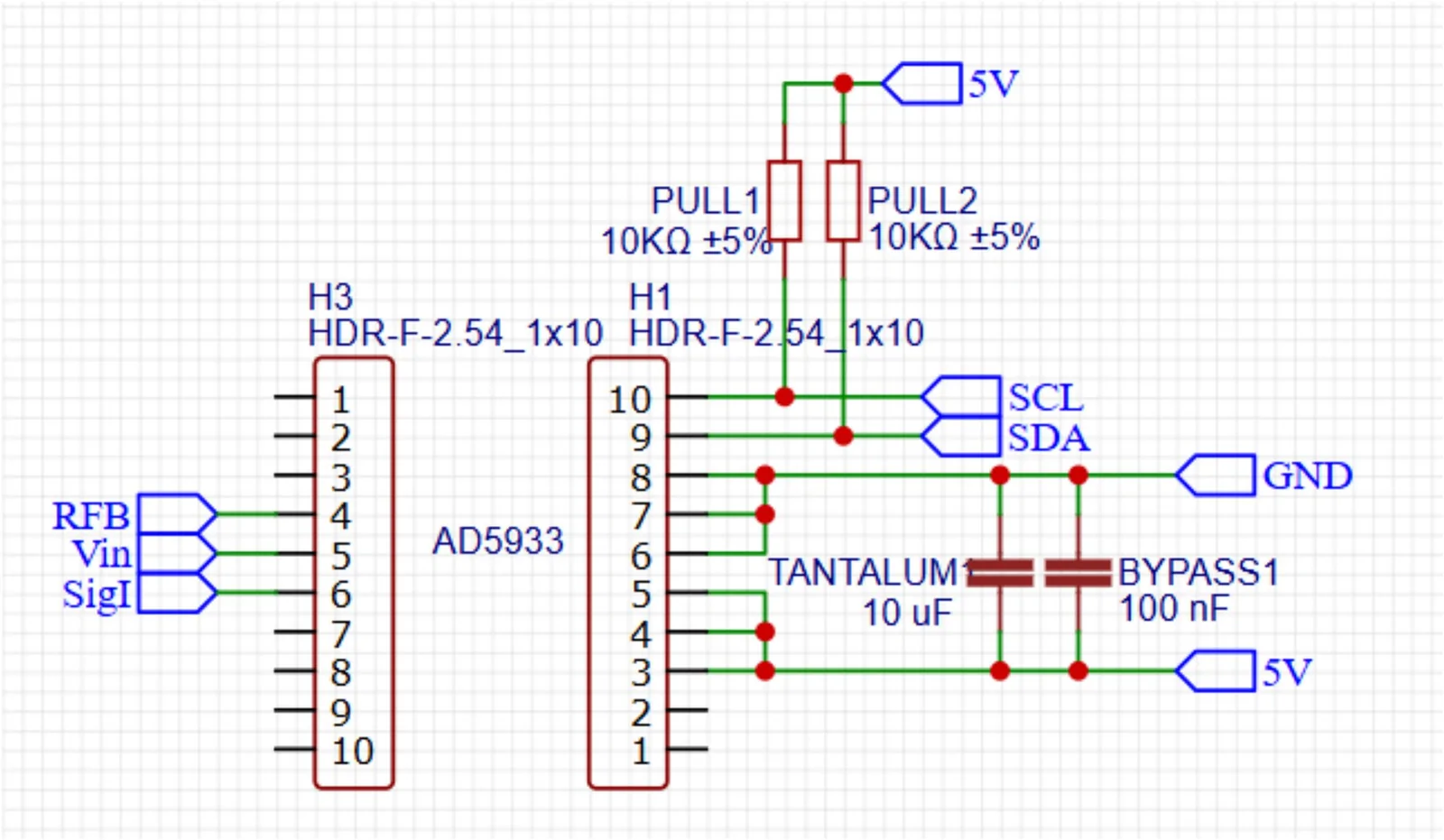
Schematic diagram of the AD5933 for signal generator and impedance converter.

In this EIT system, the AD5933 was selected as the core component because it integrates two functions within a single integrated circuit (IC): a signal generator and an impedance converter [13]. The AD5933 is utilized to generate an excitation signal for the Voltage-Controlled Current Source (VCCS) and to convert the measured boundary voltages from the instrumentation amplifier (IA) into impedance data [4]. Although the AD5933 can generate signals up to 100 kHz with an amplitude of 2 Vpp, we specifically configured it to output a 50 kHz, 1 Vpp sinusoidal voltage signal for this study. The current flowing through the impedance load is converted into a voltage and sampled by the internal Analog-to-Digital Converter (ADC). Subsequently, the on-chip Digital Signal Processor (DSP) processes both the generated and received signals using a Discrete Fourier Transform (DFT) algorithm. This process yields the real (R) and imaginary (I) parts of the complex signal at the designated discrete frequency, which are then transmitted to the microcontroller via the I2C serial interface for further impedance calculation [13]. Fig. 2 illustrates the hardware schematic of the AD5933 circuit, which uses two 10 kΩ pull-up resistors on the Serial Data (SDA) and Serial Clock (SCL) pins to ensure stable and reliable I2C communication with the microcontroller [14].

### 2.2 Microcontroller and power supply

The proposed EIT system is controlled by an STM32F407VGT6 microcontroller, which actively manages both the data acquisition sequence and serial communication. Power for the entire system is supplied directly from a personal computer (PC) via a standard USB connection. This USB interface serves a dual purpose: it acts as the primary 5 V power source and functions as the Universal Asynchronous Receiver-Transmitter (UART) data link for executing the Python-based visualization software. To accommodate the analog components requiring a bipolar supply, the incoming 5 V is directly routed to the positive power rail, while an MC34063A DC-DC converter module is employed to invert the 5 V into a stable −5 V supply for the negative power rail [7]. Furthermore, a decoupling strategy is implemented to ensure optimal power integrity and suppress high-frequency digital noise generated by the microcontroller and multiplexing operations. Specifically, a parallel combination of a 10 μF tantalum capacitor and a 100 nF ceramic capacitor is strategically placed as close as possible to the power supply pins of every active integrated circuit (IC) including the AD5933, AD620, INA118P, and TL084 across the shared power rails [15].

### 2.3 Voltage-controlled current source (VCCS)

**Fig. 3:**
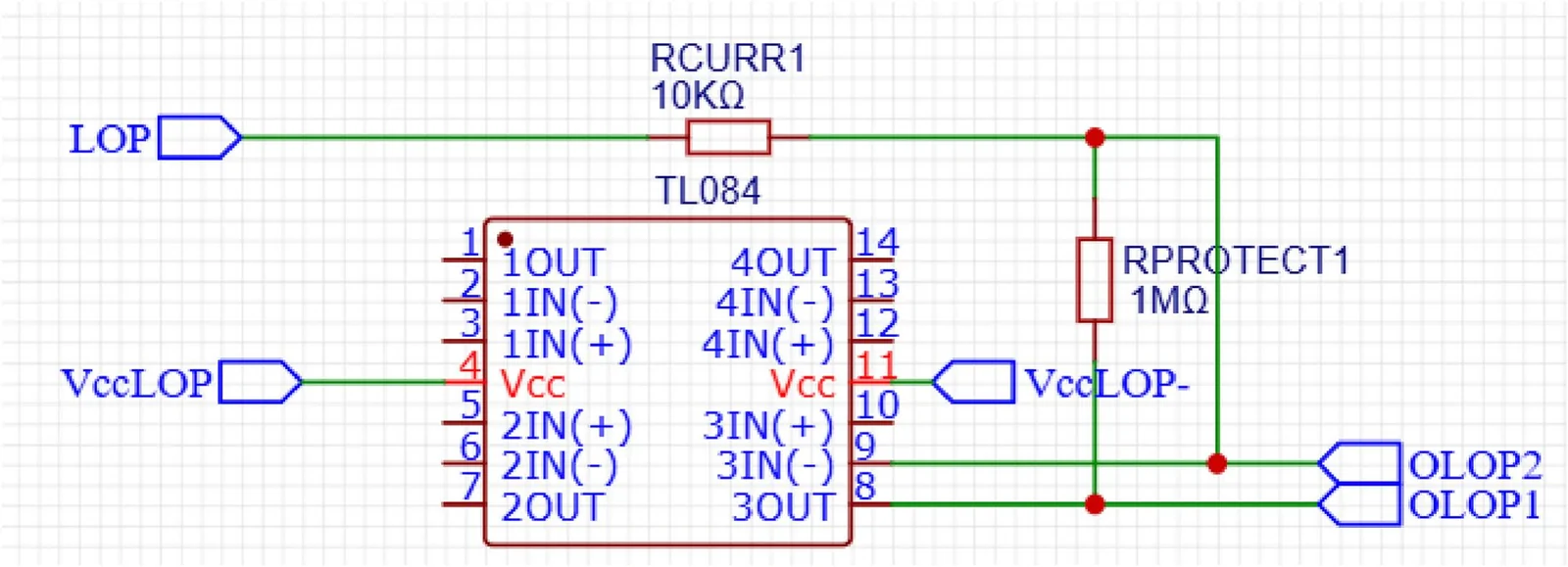
Schematic diagram of the Load-in-the-Loop Current Source (LOP) as VCCS.

**Fig. 4:**
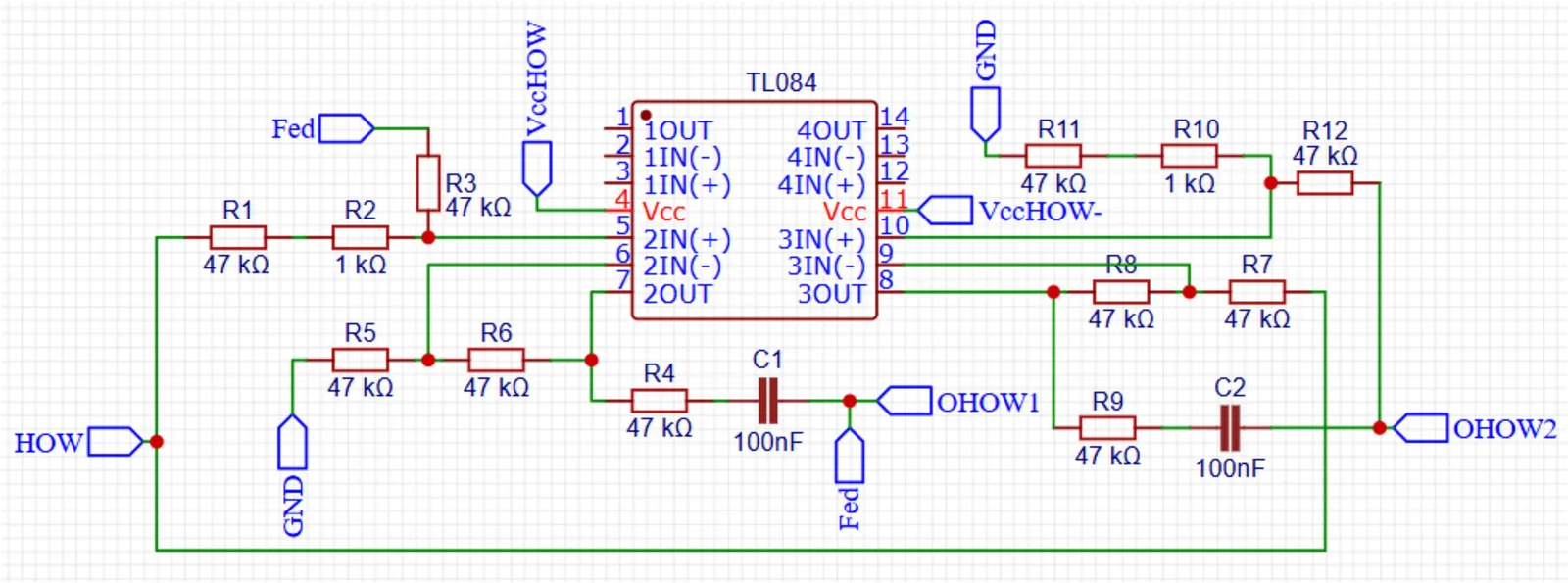
Schematic diagram of the Mirrored Howland Current Source (HOW) as VCCS.

Proposed hardware architecture offers two selectable configurations for the Voltage-Controlled Current Source (VCCS) to accommodate different experimental needs: a Load-in-the-Loop current source (LOP) and a Mirrored Howland Current Source (HOW). Fig. 3 illustrates the schematic of the Load-in-the-Loop circuit alongside the hardware switches, while Fig. 4 details the configuration of the Mirrored Howland Current Source adapted from [16]. During operation, users must configure the onboard switches to route the excitation signal through only one of these VCCS pathways to avoid electrical conflicts. Both designs utilize a 14-pin TL084CN quad operational amplifier to reliably convert the analog voltage output from the AD5933 into a constant excitation current. The TL084CN operational amplifier was selected due to its optimal balance of high-speed performance, low power consumption, and extremely high input impedance [17]. With a unity gain-bandwidth product (GBP) of 3 MHz and a high slew rate of 13 V/µs, the amplifier easily accommodates our 50 kHz operating frequency without amplitude attenuation or phase distortion. Additionally, the quad-packaged layout of the TL084CN consolidates multiple active stages into a single integrated circuit, reducing physical PCB footprint and parasitic trace capacitances.

The resistance values and detailed electronic components for the Load-in-the-Loop Current Source (Fig. 3) and the Mirrored Howland Current Source (Fig. 4) are specified within their hardware schematics. When implementing the HOW topology, utilizing precision resistors with a tolerance of 1% or less is strictly required due to the configuration's high sensitivity to component mismatch [16]. Adhering to this tight tolerance is crucial to preserve high output impedance, thereby guaranteeing a stable and accurate constant current injection across varying phantom loads.

### 2.4 Multiplexer

To distribute the excitation current and measure the resulting boundary voltages across the 16-electrode array, the system utilizes four 16-channel CD74HC4067 multiplexers. These multiplexers dynamically route the current injected by the VCCS and the voltage measurements acquired by the instrumentation amplifier (IA). The switching architecture is explicitly designed to support the four-terminal sensing method using an adjacent common pattern. In this configuration, two neighboring electrodes are assigned to inject the current, while another distinct adjacent pair is used to measure the potential difference across the boundary.

**Fig. 5:**
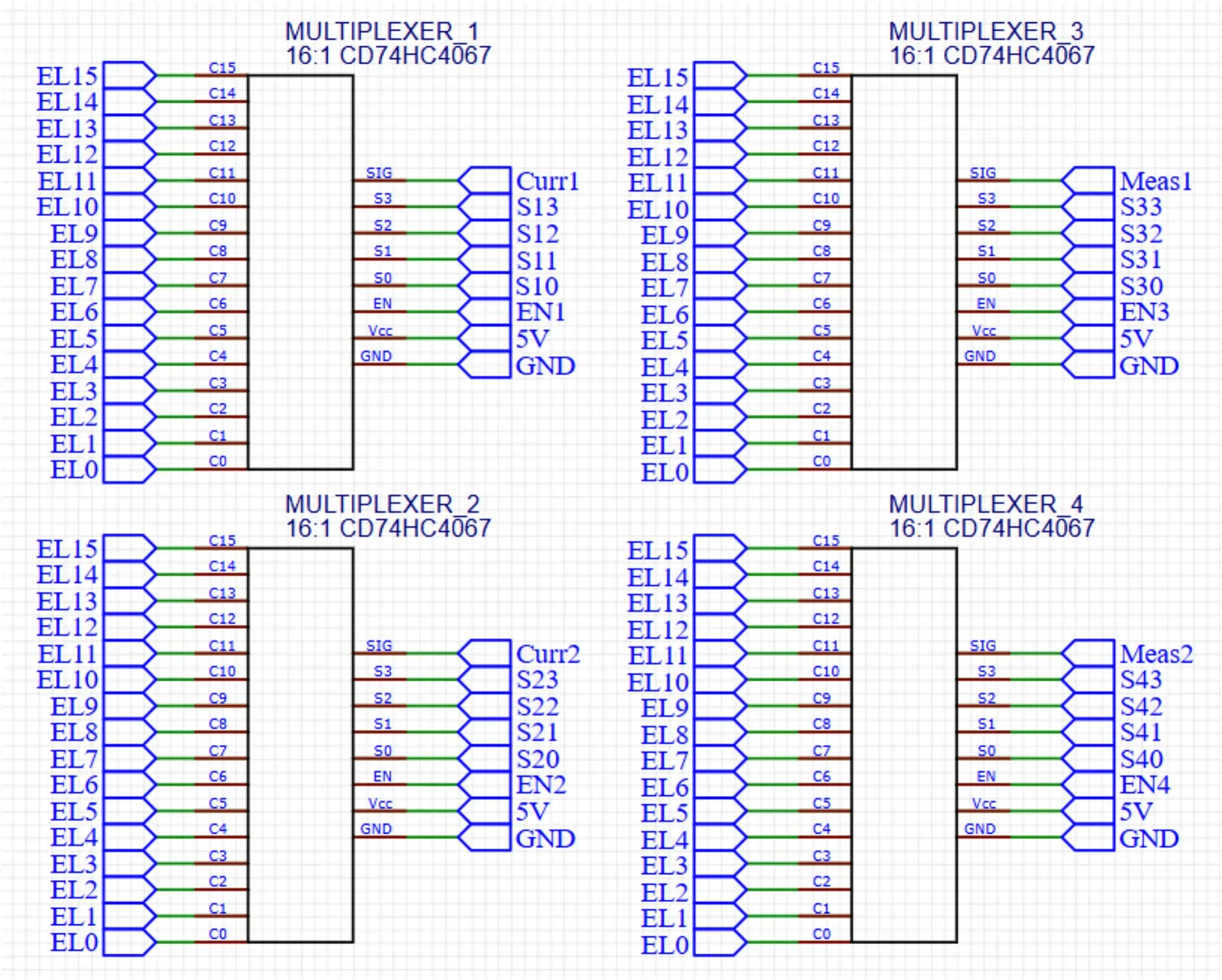
16-channel multiplexer array for signal routing.

As illustrated in Fig. 5, the signal routing mechanism of each multiplexer controlled by a 4-bit selection pins S0 to S3 managed by the microcontroller. To activate the routing path, the microcontroller must assert the Enable (EN) pin to turn on the corresponding multiplexer. Once enabled, the specific 4-bit binary sequence dictates which of the 16 independent channels is connected to the common signal line (SIG) [18]. This SIG pin acts as the primary bidirectional conduit, directly linking the core analog circuits either the VCCS output or the instrumentation amplifier (IA) inputs to the physical electrodes attached to the phantom.

Although the CD74HC4067 multiplexer introduces parasitic capacitances and non-zero resistance on each channel, the impacts on the measurement accuracy are bypassed by the image reconstruction algorithm. This EIT system operates on time-difference EIT, any static hardware-induced offsets including baseline parasitic capacitances and multiplexer channel resistances across all 16 channels are mathematically subtracted and cancelled during the differential image reconstruction process.

**Fig. 6:**
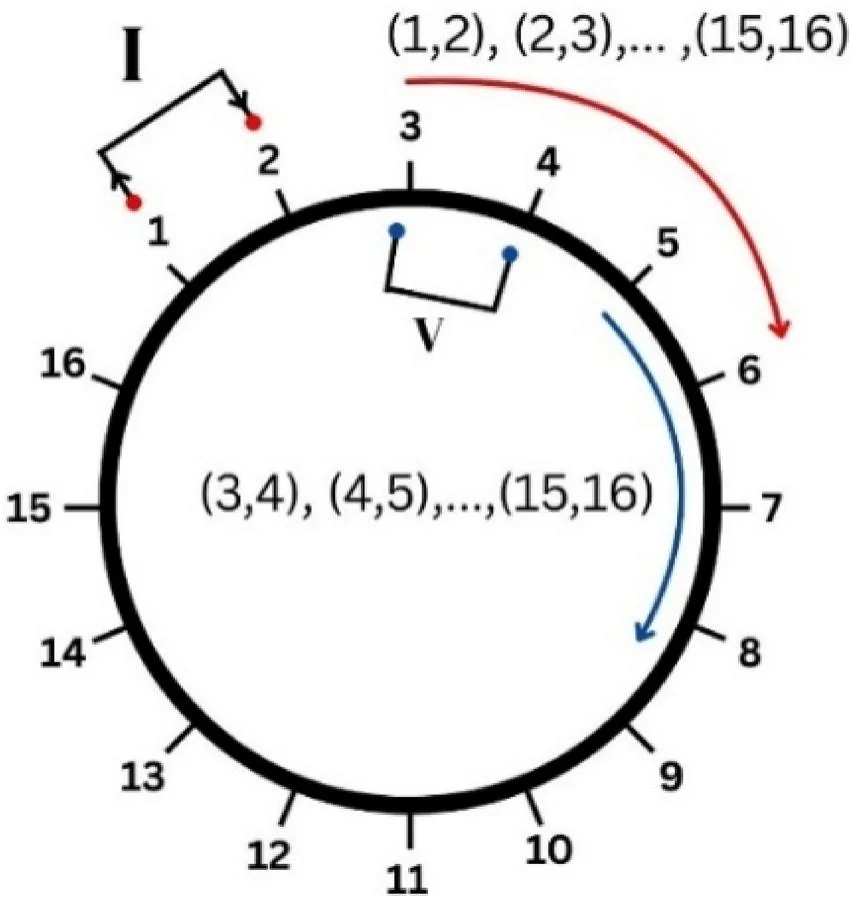
Adjacent switching method for 16-electrode impedance data acquisition.

To systematically acquire this data, the process begins by injecting a constant alternating current through the first pair of neighboring electrodes, designated as electrodes 1 and 2. While this current is applied, the resulting differential voltages are sequentially measured across all remaining non-injecting adjacent electrode pairs, starting from electrodes 3 and 4, followed by 4 and 5, and continuing up to electrodes 15 and 16. Once a full sweep of voltage measurements is completed for this initial injection site, the microcontroller directs the multiplexers to shift the active excitation channels to the next consecutive adjacent pair, namely electrodes 2 and 3. The voltage measurement sequence is then systematically repeated for this new injection pair. As illustrated in Fig. 6, this circular shifting of injection and measurement roles around the phantom object ensures comprehensive data collection, generating a total of 208 impedance data for the image reconstruction process [9].

Although injection patterns, such as opposite or diagonal (cross) switching, are theoretically capable of distributing current more deeply into the domain to reduce far-channel noise, our comparative tests demonstrated that the adjacent pattern remains superior for accurately reconstructing the shape and location of inclusions in our setup. While adjacent injection is more susceptible to localized boundary artifacts near the container edge due to high peripheral current density. Cross and opposite patterns introduced excessive measurement noise so that the resulting image failed to detect objects inside the container.

### 2.5 Instrumentation amplifier

**Fig. 7:**
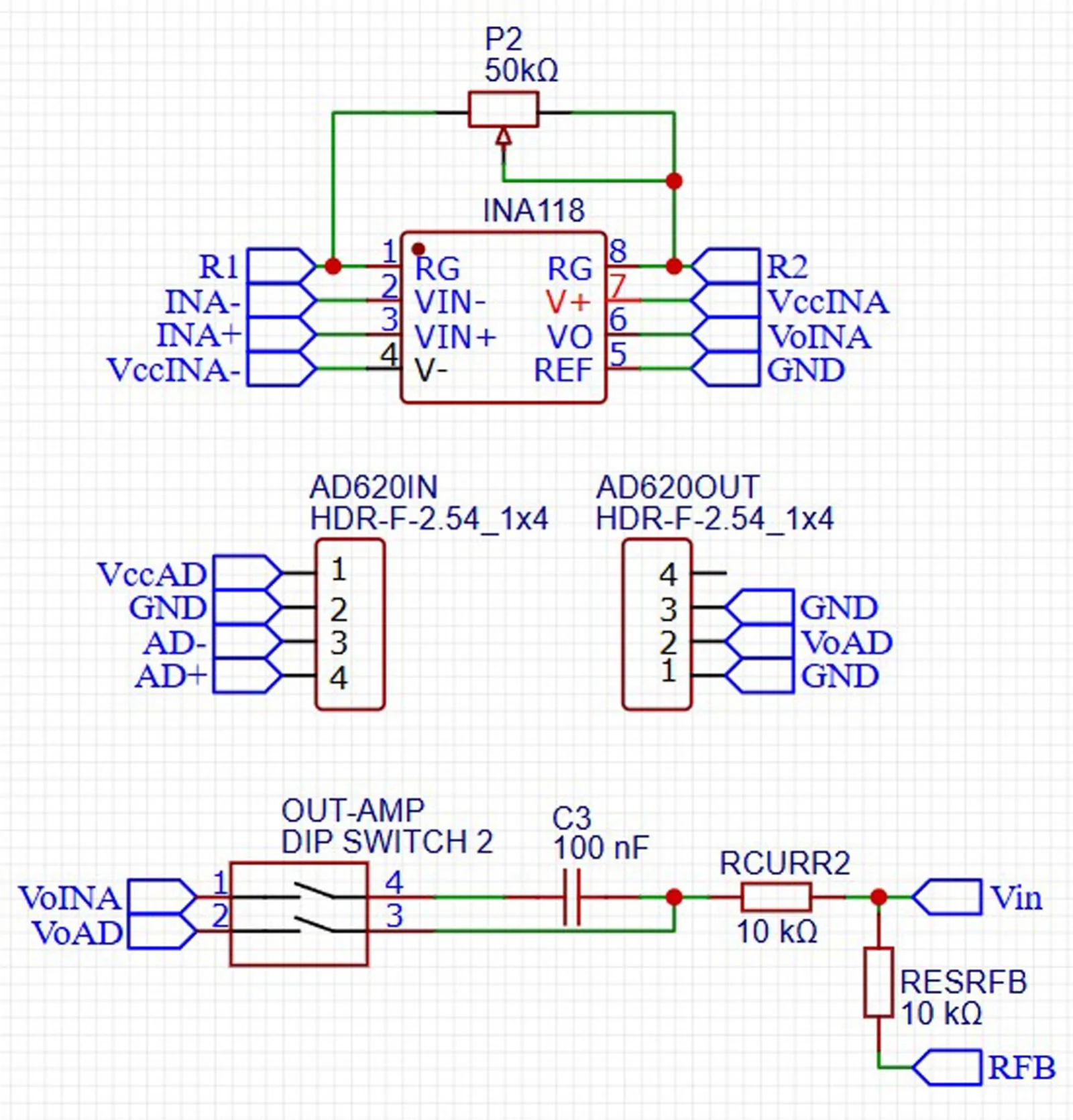
INA118P and AD620 instrumentation amplifier (IA) for differential voltage amplification.

Capturing the weak differential voltage signals from the measuring electrodes requires robust amplification. As depicted in Fig. 7, the system supports either a discrete INA118P IC or AD620 module. This dual compatibility maximizes reproducibility by allowing users to select the amplifier that best fits their local component availability and specific research needs. To facilitate signal calibration, the INA118P circuit has a 50 kΩ trimpot for variable gain control, while the AD620 module features built-in trimpots for both gain and DC offset adjustments [11]. While this offset tuning enables single-supply operation to prevent negative signal clipping, it inherently restricts the output voltage headroom. Conversely, the INA118 IC requires a dual-polarity power supply, which provides a wider dynamic range for AC amplification without DC bias. Furthermore, although the AD620 exhibits a higher operational bandwidth, the INA118P consumes substantially less quiescent current, making it highly efficient for low-power portable applications. Both options deliver the high common-mode rejection and signal amplification required to multiply the boundary voltages to optimal level [19].

Following this amplification stage, the conditioned voltage output is routed through a series resistor, designated as RCURR2, before being fed directly into the pin of the AD5933 [16]. This specific series resistor acts as a voltage-to-current converter, ensuring the amplified signal is appropriately formatted as a current input for the AD5933′s internal transimpedance amplifier (TIA). To complete this standard analog front-end configuration, a feedback resistor (REDRFB) is externally connected in parallel between the and RFB pins of the AD5933 [13]. This resistor works with the internal TIA to form a robust current-to-voltage gain stage. Consequently, by carefully tailoring the ratio between and RCURR2, users can adjust the internal system gain to utilize the range of the internal analog-to-digital converter (ADC) while strictly preventing signal clipping or saturation during data acquisition [20].

### 2.6 Electrodes and experiment container

**Fig. 8:**
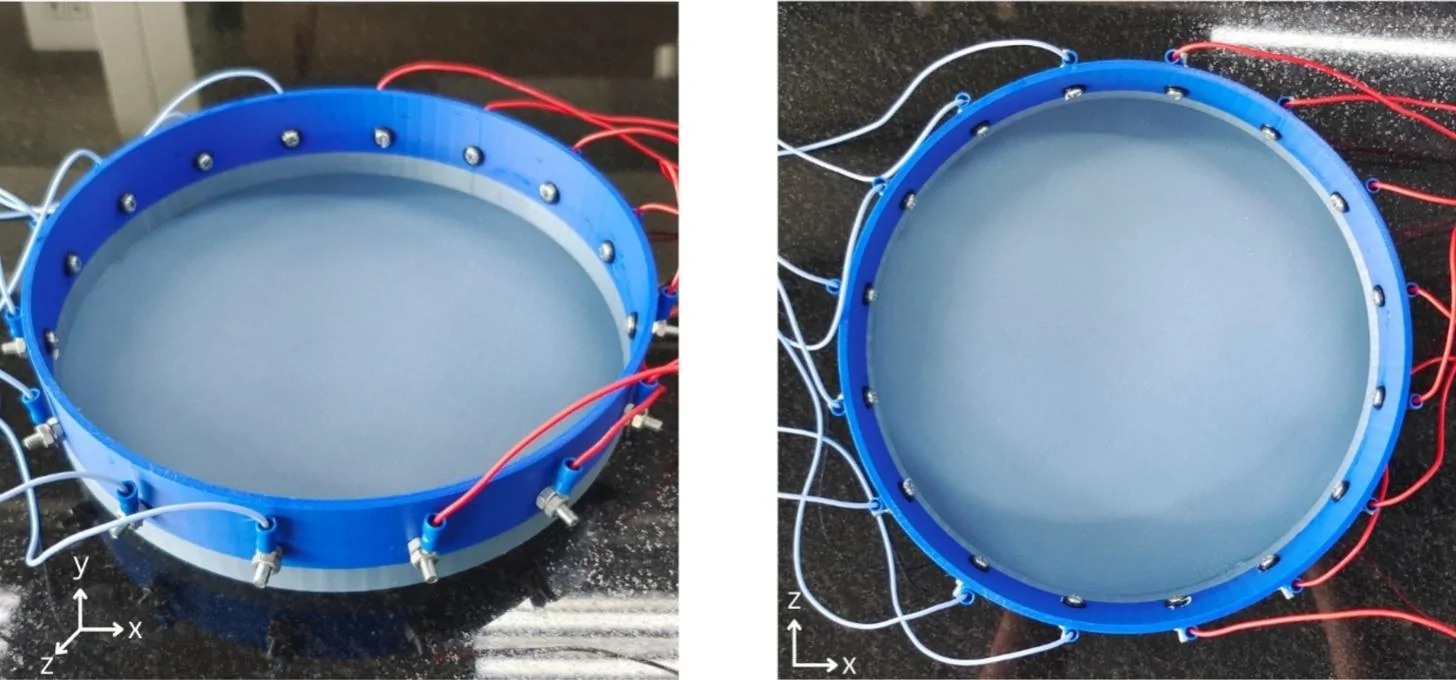
The 3D-printed PLA container equipped with 16 electrodes. Rubber rings are clamped inside the container, and PTFE tape wrapped around the threads of the stainless steel bolts to ensure a watertight seal.

Fig. 8 illustrates the 3D-printed phantom container with its 16-electrode configuration used for the experimental validation. The container is fabricated from Polylactic Acid (PLA) and features a compact cylindrical geometry with a diameter of 10 cm and a height of 4 cm [5]. To form the boundary sensing interface, 16 stainless steel bolts are utilized as electrodes, positioned equidistantly around the container's perimeter at a height of 2 cm to establish a precise mid-plane measurement cross-section [21]. Given that the target medium inside the phantom is aqueous, robust waterproofing is strictly implemented to prevent conductive fluid leakage that could short-circuit the system or alter the impedance environment. Specifically, rubber O-rings are placed against the inner wall of the container, and white polytetrafluoroethylene (PTFE) seal tape is tightly wrapped around the bolt threads prior to installation. For reliable electrical interfacing, jumper wires are soldered to Y-shaped fork connectors, which are then firmly clamped to the external threaded portion of each bolt using secure nuts. This mechanical connection ensures a stable electrode contact and minimizes signal degradation during data acquisition.

To highlight the broader utility of this platform, the following points summarize how the proposed hardware can assist researchers and educators in performing both standard and novel laboratory tasks:

• The modular analog front-end enables researchers to physically switch between dual current source topologies and dual instrumentation amplifiers on a single PCB, facilitating direct empirical comparisons of different circuit designs under identical operating conditions.
• Operating at a replication cost under 100 USD with standard through-hole components, this platform provides an affordable and easily reproducible EIT testbed for researchers.
• The inclusion of IC sockets and a modular PCB layout allow hardware developers to easily swap active components, modify feedback networks, and test custom analog circuit blocks without fabricating a new board.
• Integrated with Python and the pyEIT library, the system provides software developers with a practical platform to validate and benchmark 2D image reconstruction algorithms using real, noisy experimental data instead of computer simulations.
• The editable Autodesk Fusion 360 (.f3d) design files allow researchers to study the CAD modeling methodology of the experiment container and customize its physical dimensions, volume, or electrode positions to suit their specific experimental requirements.

## 3 Design files summary

In this study, Python version 3.12.0 and pyEIT version 1.2.4 were utilized to develop the system and execute the experimental evaluations. Summary of the design files, including the hardware schematics, PCB layouts, and software components necessary to replicate this system, is presented in the Table 2.

• **EIT Image Reconstruction and GUI:** This Python script utilizes the open-source pyEIT library to reconstruct conductivity images from impedance data [22]. It also features a Tkinter-based Graphical User Interface (GUI) to facilitate data acquisition, file management, and visualization.
• **EIT-STM32 Circuit Schematic-EasyEDA:** This file contains the complete electronic schematic of the EIT system designed in the EasyEDA software. It details the wiring and integration of the microcontroller, AD5933, voltage-controlled current source (VCCS), multiplexer arrays, instrumentation amplifier (IA), switches, and power supply configuration.
• **EIT-STM32 PCB Gerber File:** This ZIP archive provides the standard computer-aided manufacturing (CAM) files required by commercial fabrication services to manufacture the custom printed circuit board (PCB).
• **AD5933_Calibration:** This project folder contains the STM32 microcontroller firmware necessary to calibrate the AD5933 prior to the data collection.
• **Data_Acquisition:** This project folder includes the firmware responsible for executing the adjacent four-terminal sensing method. It actively manages the sequential switching of the multiplexers and transmits the measured impedance data to the personal computer via UART serial communication.
• **Container-EIT:** This STL file provides the 3D printable model for the 20 cm diameter phantom container. It is designed to be printed using Polylactic Acid (PLA) and includes placements for installing the 16 electrode stainless steel bolts.

**Table 2:** Design File Summary.

**Design file name**	**File type**	**Open source license**	**Location of the file**	**File description**
EIT Image Reconstruction and GUI	.py file	MIT License	Available with the article	Firmware
EIT-STM32 Circuit Schematic-EasyEDA	.json file	CERN-OHL	Available with the article	Schematic
EIT-STM32 PCB Gerber File	.zip file	CERN-OHL	Available with the article	PCB Gerber Files
AD5933_Calibration	Project folder	MIT License	Available with the article	Firmware
Data_Acquisition	Project folder	MIT License	Available with the article	Firmware
Container-EIT	.stl file	CC BY 4.0	Available with the article	File for 3D print
Calibration and data acquisition guide	.mp4	CC BY 4.0	Available with the article	Tutorial

## 4 Bill of materials summary

The bill of materials required for the fabrication and assembly of the EIT system is summarized in Table 3.

**Table 3:** Bill of Materials.

**Designator**	**Component**	**Number**	**Cost per unit −currency**	**Total cost −** **currency**	**Source of materials**	**Material type**
Microcontroller	STM32F407VGT6 development board	1	12.88 USD	12.88 USD	link	Other
Debugger	ST-Link Debugger V.2	1	5.11 USD	5.11 USD	link	Other
Impedance Converter	AD5933	1	23.87 USD	23.87 USD	link	Other
Adapter Board	PCB Board Converter SSOP20 to 2.54 mm DIP	1	0.177 USD	0.177 USD	link	Other
Connector	8-Pin Screw Terminal Block	2	0.657 USD	1.314 USD	link	Other
IC Socket	14-Pin IC Socket	2	0.138 USD	0.276 USD	link	Other
Operational Amplifier	TL084 Op-Amp	2	0.85 USD	1.7 USD	link	Other
IC Socket	8-Pin IC Socket	1	0.134 USD	0.134 USD	link	Other
Instrumentation Amplifier	INA118P	1	16.8 USD	16.8 USD	link	Other
Instrumentation Amplifier	AD620 module	1	2.16 USD	2.16 USD	link	Other
Variable Resistor	50 kΩ Potentiometer Trimpot	1	0.162 USD	0.162 USD	link	Other
Capacitor	10 uF Tantalum Capacitor	9	0.192 USD	1.728 USD	link	Other
Capacitor	100 nF Capacitor	11	0.012 USD	0.132 USD	link	Ceramic
Multiplexer	CD74HC4067 16-Channel Multiplexer	4	1.08 USD	4.32 USD	link	Other
Resistor	1 MΩ Resistor	1	0.0136 USD	0.0136 USD	link	Other
Resistor	47 kΩ Resistor	10	0.0138 USD	0.138 USD	link	Other
Resistor	10 kΩ Resistor	4	0.0139 USD	0.0556 USD	link	Other
Resistor	1 kΩ Resistor	3	0.0138 USD	0.0414 USD	link	Other
Printed Circuit Board (PCB)	PCB Board Manufacture	1	15.1 USD	15.1 USD	link	Other
Electrode	Stainless steel screw nut set M3 20 mm	16	0.322 USD	5.152 USD	link	Metal
Electrode	Y Shape SV1.25–3 Type Fork	16	0.0225 USD	0.36 USD	link	Metal
Electrode	Rubber O-ring OD 7 mm CS 2 mm	16	0.0482 USD	0.7712 USD	link	Other
Electrode	PTFE thread seal tape	1 roll	0.82 USD	0.82 USD	link	Other
Power Supply	MC34063A Positive Voltage to Negative Voltage Module	1	2.04 USD	2.04 USD	link	Other

## 5 Build instructions

### 5.1 PCB fabrication and assembly

The main printed circuit board (PCB) can be manufactured by JLCPCB using the Gerber files provided in the design repository. Although a pick-and-place file is not provided as the original prototype was self-assembled, users can opt for a commercial PCBA (Printed Circuit Board Assembly) service since all required components are standard and explicitly listed in the Bill of Materials (BOM) along with their purchase links. For manual assembly, it is recommended to solder the lowest-profile components first, such as through-hole resistors and SMD capacitors, followed by the IC sockets, female headers, and finally the circuit modules, such as multiplexers, MC34063A, microcontroller and AD5933.

### 5.2 Hardware calibration

**Fig. 9:**
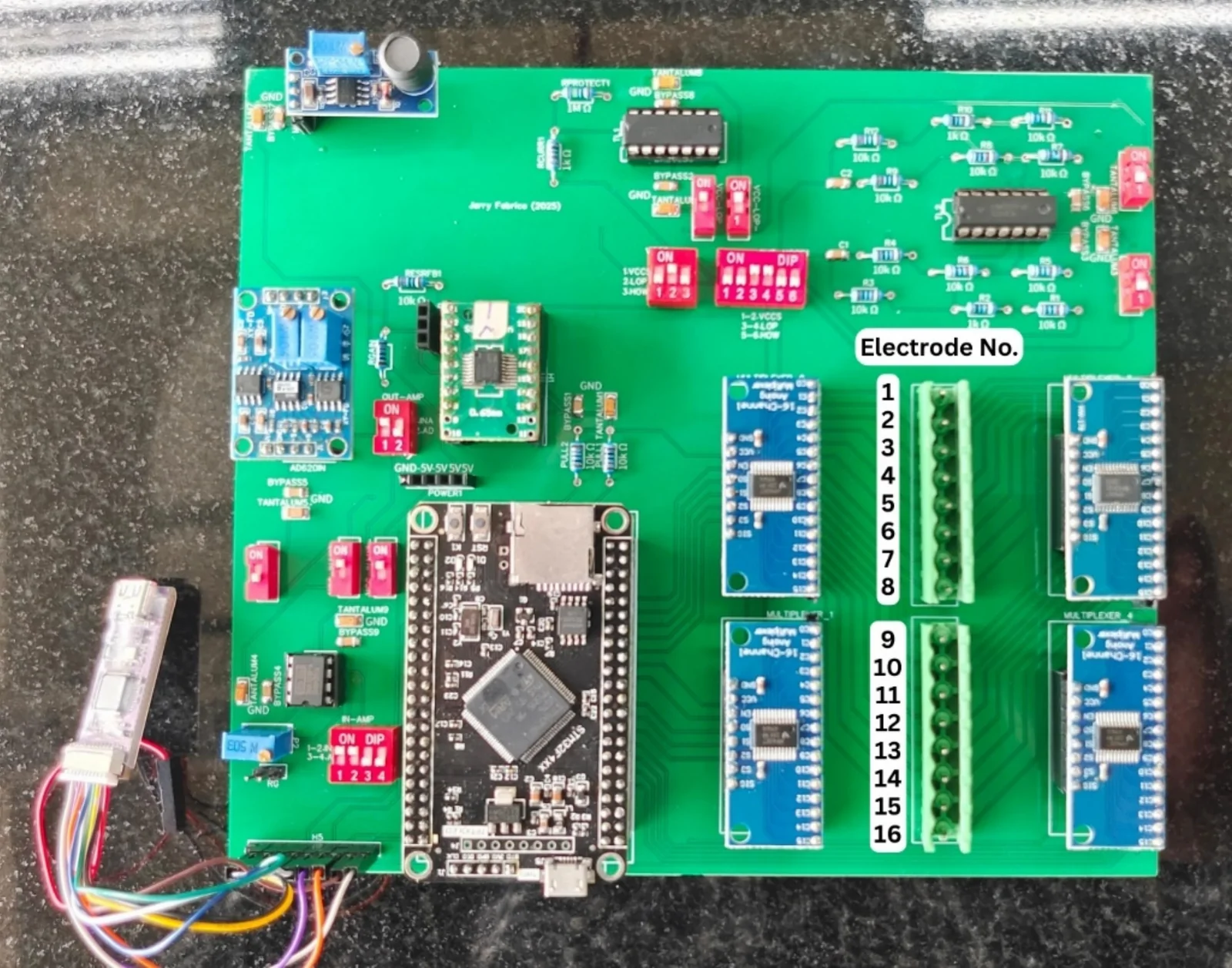
Fully assembled proposed EIT system PCB.

Before initiating the impedance measurement, initial hardware calibration is required to ensure proper signal amplification and power integrity. First, adjust the 50 kΩ trimpot potentiometer (P2) to its maximum resistance. This can be verified by connecting a digital multimeter to the H2 header pins on the PCB. Next, the bipolar power supply must be calibrated. The system utilizes an MC34063A module to invert the 5 V USB input into a −5 V rail. Using a screwdriver, carefully turn the potentiometer on the MC34063A module until a stable −5 V output is achieved. This operating voltage can be continuously monitored by placing the multimeter probes on the POWER1 header, which breaks out the GND, 5 V, and −5 V connection pins. When connecting the STM32F407VGT6 to the WeAct V2 debugger via the H5 header, only connect either the 3.3 V or the 5 V power pin. Never connect both at the same time to avoid damaging the microcontroller or the debugger. Fig. 9 depicts a fully assembled PCB of the proposed EIT system.

### 5.3 Container and electrodes fabrication

The phantom container is manufactured using a 3D printer with Polylactic Acid (PLA) filament. The 3D model (.stl format) can be downloaded from the design repository. Once printed, construct the electrode array by installing the 16 M3 stainless steel bolts into the pre-designed holes along the container's perimeter. To prevent saline solution leakage, wrap the bolt threads with white PTFE seal tape and insert rubber O-rings on the inner wall before tightening them. For the electrical interfacing, solder jumper wires from the multiplexer outputs to Y-shape fork terminals, and secure them tightly to the external side of each bolt using nuts (see Fig. 8).

### 5.4 Firmware and software installation

The final step involves setting up the software and firmware environment. First, install STM32CubeIDE version 1.18.0 to compile and flash the microcontroller firmware onto the STM32F407VGT6 development board via the debugger. For data acquisition and image reconstruction, install Visual Studio Code (VSCode) or a similar Python IDE on the personal computer. Ensure that Python (version 3.12.0) is installed along with all the necessary dependencies required to execute the visualization code. Specifically, users must install the open-source pyEIT framework (version 1.2.4) to handle the meshing procedures and execute the BP, JAC, and GREIT reconstruction algorithms [22]. Additional required libraries include pyserial for UART communication with the microcontroller, matplotlib for rendering the EIT graphs, as well as numpy and pandas for numerical data operations. The code also relies on Python's built-in standard libraries, namely tkinter for the Graphical User Interface (GUI), os, and time. Once the firmware is flashed and all Python dependencies are successfully installed, the hardware can be connected to the PC via a USB cable to begin automated data acquisition.

## 6 Operation instructions

To ensure safe and proper operation of the proposed EIT system, users must strictly follow the hardware configuration and software execution steps outlined below.

### 6.1 Hardware configuration

Prior to initiating the data acquisition, the hardware must be appropriately configured by setting the red DIP switches located on the fully assembled PCB (see Fig. 9). As established in the hardware design, the system provides two VCCS options (Load-in-the-Loop and Mirrored Howland) and two instrumentation amplifier options (INA118P and AD620). To minimize electrical noise and prevent unwanted interference, only the power supply lines (positive and negative rails) of the actively utilized circuits must be turned on. Although the switch routing numbers are clearly printed on the PCB surface for convenience, users must carefully configure the switches according to the truth table provided in Table 4 to match their desired experimental setup.

**Table 4:** Switch Truthtable.

Configuration Mode	VCCS (Pin 2)	VCCS (Pin 3)	Out VCCS (Pins 3 & 4)	Out VCCS (Pins 5 & 6)	IN AMP (Pins 1 & 2)	IN AMP (Pins 3 & 4)
1. LOP & INA118P Active	ON	OFF	ON	OFF	ON	OFF
2. LOP & AD620 Active	ON	OFF	ON	OFF	OFF	ON
3. Howland & INA118P Active	OFF	ON	OFF	ON	ON	OFF
4. Howland & AD620 Active	OFF	ON	OFF	ON	OFF	ON
5. Standby / All OFF	OFF	OFF	OFF	OFF	OFF	OFF
6. INVALID (Conflict)	ON	ON	ON	ON	ON	ON

Safety Note: Extreme caution must be taken to never configure the switches into Mode 6 (INVALID). Turning all switches ON simultaneously will cause a severe electrical conflict between the dual VCCS and instrumentation amplifier circuits, potentially leading to short circuits and permanent hardware damage.

### 6.2 Firmware flashing

Once the hardware is configured, the microcontroller must be programmed.

1. Ensure that STM32CubeIDE (version 1.18.0) is installed on your computer.
2. Import the provided AD5933_Calibration and Data_Acquisition project folders from the design repository into your workspace.
3. To import, navigate to File > Import > General > Existing Projects into Workspace, and click Next.
4. Choose Select archive file, browse for the downloaded EIT project files, and click Finish.
5. Compile and flash the respective firmware to the STM32 microcontroller via the debugger.

### 6.3 Gain factor calibration

Before initiating the actual data acquisition, users must calibrate the AD5933 impedance converter to determine the system's specific gain factor. This procedure ensures accurate impedance calculations and must be strictly repeated whenever the background medium inside the phantom container is altered.

1. Project Initialization: Open STM32CubeIDE, load the AD5933_Calibration project into the workspace, and open the main source code located at Core/Src/main.c.
2. Execution: Click the Debug icon to compile and flash the calibration firmware onto the microcontroller.
3. Monitoring: Enable the ITM Data Console within the IDE to monitor the serial data stream.
4. Recording: Observe the console output and carefully record the calculated gain factor value displayed on the screen.
5. Applying the Calibration: Open the main Data_Acquisition project file. Navigate to line 60 of the main source code and update the gain_factor variable with the newly acquired value.
6. Finalizing: Save the changes, recompile, and flash the updated Data_Acquisition firmware to the microcontroller to proceed with the experiments.

For further clarity and a more detailed visual guide, a comprehensive video tutorial demonstrating this step-by-step calibration procedure has been uploaded to the design file repository.

### 6.4 Data acquisition and image reconstruction (GUI operation)

The final operational phase involves executing the Python-based visualization software to automate the data acquisition process.

1. Software Initialization: Open the EIT Image Reconstruction and GUI.py file using a Python-compatible code editor, such as Visual Studio Code or Spyder.
2. Launching the Interface: Run the Python script to launch the custom pyEIT-based Graphical User Interface (GUI), as illustrated in Fig. 10.

**Fig. 10:**
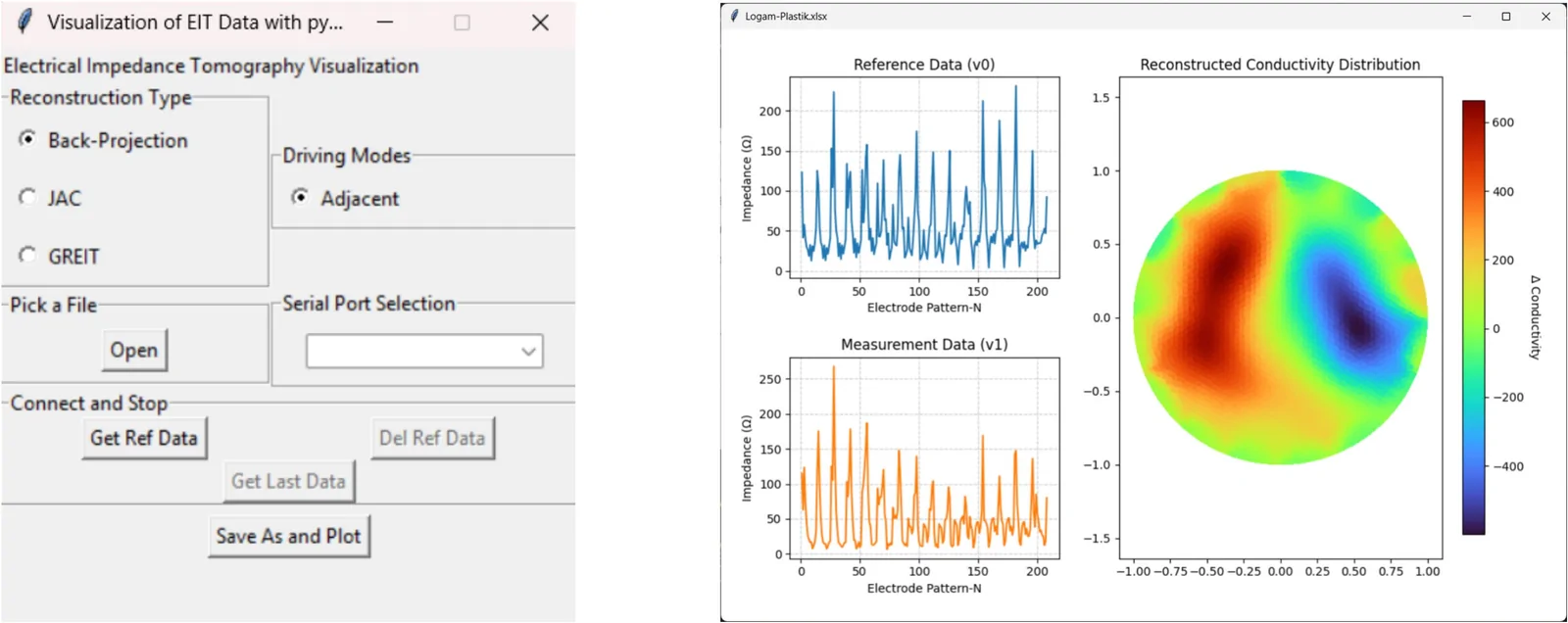
Custom graphical user interface for data acquisition and reconstruction.
3. Algorithm Selection: In the GUI, select the desired image reconstruction algorithm from the available buttons, which include Back-Projection, JAC, and GREIT.
4. Port Connection: Select the COM port connected to the STM32 microcontroller from the 'Serial Port Selection' dropdown menu. The correct COM port number can be verified by identifying the STM32 Virtual COM Port within the Windows Device Manager.
5. Baseline Measurement: Ensure the phantom container is filled only with the homogeneous background medium. Click the 'Get Ref Data' button to acquire the reference impedance data.
6. Anomaly Measurement: Insert the target phantom object into the container. Click the 'Get Last Data' button to acquire the new impedance data reflecting the anomaly.
7. Visualization: Click the 'Save as and plot' button. The software will automatically save the experimental data to an Excel file and render the 2D reconstructed EIT image alongside the measured voltage graphs.
8. Continuous Monitoring: Users can continuously iterate the 'Get Last Data' and 'Save as and plot' actions to capture and save multiple frames of moving objects using the same reference impedance data.
9. Resetting Data: To conduct a new experiment with a different background medium, click the 'Del Ref Data' button to clear the current reference data from the system's memory.

## 7 Validation and characterization

This chapter outlines the experimental conditions and methodologies applied throughout the evaluation of the proposed EIT hardware. Initially, the excitation signals generated by the voltage-controlled current source (VCCS) were directly observed and validated using an oscilloscope. Subsequently, the Signal-to-Noise Ratio (SNR) of the system was evaluated under two distinct conditions: an empty container and a container filled with a saline water medium possessing a measured conductivity of 966 mS/m at 25°C. For robust statistical analysis, the SNR data was extracted from 50 consecutive measurement acquisitions. Furthermore, to evaluate the absolute hardware accuracy and repeatability using standard resistors, the two-terminal resistive loads were connected directly to channels 1 and 2. Consequently, both the current injection and voltage measurement routing were exclusively configured to these two channels, with measurements repeated 50 times for each evaluated resistor value. Finally, the practical image reconstruction capability of the system was tested inside the tank container filled with the 966 mS/m at 25°C saline medium. Two cylindrical phantom tubes one metallic (highly conductive) and one plastic (non-conductive) were utilized as targets to introduce extreme conductivity contrasts within the background domain. The detailed results and comprehensive analyses for each of these experimental validations are presented in the following subsections.

### 7.1 Excitation signal characterization

**Fig. 11:**
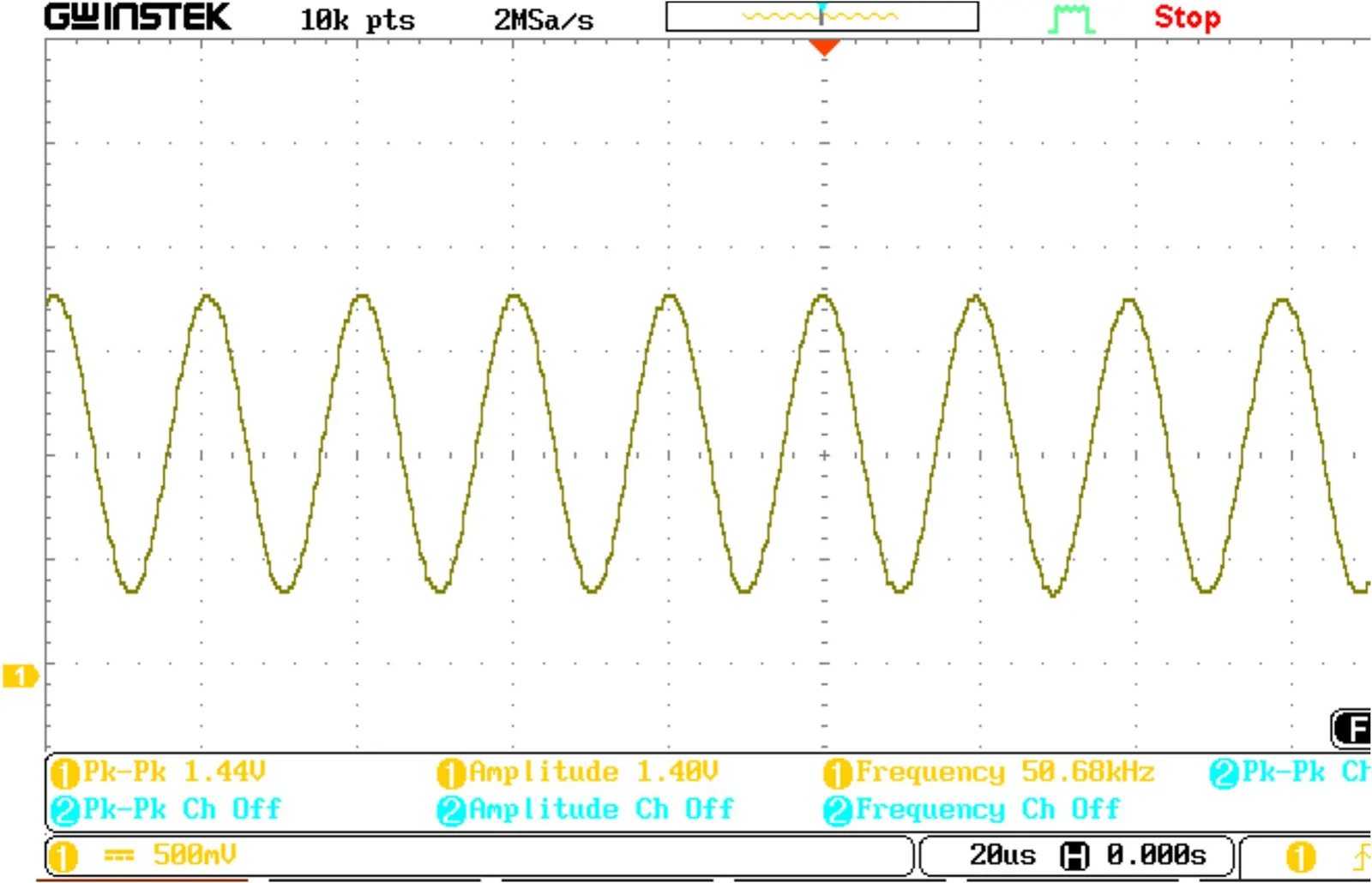
Signal on AD5933 output before going into VCCS.

The AD5933 impedance converter was configured to generate a nominal sinusoidal excitation signal of 50 kHz with a peak-to-peak amplitude of 1 Vpp. However, as depicted by the oscilloscope measurement in Fig. 11, the actual generated output signal exhibited a precise frequency of 50.68 kHz and an amplitude of 1.44 Vpp. This slight deviation is a standard hardware behavior; the 0.68 kHz frequency offset is attributed to the internal DDS quantization and crystal oscillator tolerance, while the higher 1.44 Vpp amplitude is the expected ratiometric scaling result of powering the AD5933 with a 5 V supply instead of the typical 3.3 V.

To ensure a reliable constant current output, this voltage signal is fed into a load-in-the-loop VCCS circuit based on a TL084 operational amplifier. In this topology, the magnitude of the alternating current injected into the phantom object is directly determined by as RCURR1, which is connected to the inverting pin of the op-amp. As derived from Fig. 3, the theoretical output current can be calculated using Equation (1), assuming an ideal TL084 operational amplifier:

(1) $$I_{\mathrm{out}}=\frac{V_{\mathrm{in}}}{RCURR1}$$

$$I_{\mathrm{out}}=\frac{1.44V}{1k\Omega }=1.44mA_{pp}$$

Alternatively, when utilizing the modified Howland current pump topology in Fig. 4, the stability of the generated constant current relies on the precise symmetry of the resistor matrix within its differential amplifier configuration. Provided that these operational amplifier resistors are perfectly matched to establish a unity gain, the current calculation simplifies significantly. Under unity gain condition, the injected current becomes solely dependent on one specific sensing resistor designated as R2 in the schematic, and can be calculated using Equation (2), assuming an ideal TL084 operational amplifier:

(2) $$I_{\mathrm{out}}=\frac{V_{\mathrm{in}}}{R2}$$

$$I_{\mathrm{out}}=\frac{1.44V}{1k\Omega }=1.44mA_{pp}$$

where $V_{\mathrm{in}}$ is the output voltage from the AD5933 (V), $I_{\mathrm{out}}$ is the resulting peak-to-peak excitation current (A)[23], RCURR1(Ω) and R2(Ω) are the current-setting and sensing resistors, respectively.

### 7.2 Gain measurement on instrumentation amplifier

Capturing the weak differential voltage signals from the measuring electrodes requires robust amplification. As depicted in Fig. 7, the system supports either a discrete INA118P IC or an AD620 module. To ensure the amplified signals remain within the optimal dynamic range of the AD5933 without clipping, the amplification gain (G) for both options is uniformly configured to approximately 2. When utilizing the INA118P circuit, the onboard 50 k trimpot is adjusted to its resistance ($R_{g}$) to achieve this target gain, as governed by using Equation (3):

(3) $$G=\;1+\frac{50\;k\Omega }{R_{g}}$$

Alternatively, when employing the AD620 module, its trimpot is precisely tuned to yield an equivalent gain of 2, according to its characteristic on Equation (4):

(4) $$G=\;1+\frac{49.4k\Omega }{R_{g}}$$

where G is the signal gain (−), and $R_{g}$ is the resistance value of the potentiometer trimpot (Ω).

Beyond signal amplification, this hardware stage is designed to regulate the output signal's DC bias voltage. Because the AD5933 utilizes a unipolar ADC, it strictly requires a biased input to prevent negative signal clipping and ensure accurate impedance measurements. To achieve this, the system employs two distinct DC-biasing mechanisms depending on the selected amplifier. When utilizing the pre-assembled AD620 module, the DC bias is manually regulated by tuning its built-in offset potentiometer to shift the AC signal into a fully positive voltage range. Conversely, when the discrete INA118P IC is employed, a 100 nF ceramic capacitor is inserted in series at the output stage. This capacitor acts as a DC-blocking component. By completely isolating the output DC level of the INA118P, the pure AC signal is allowed to pass through and naturally ride on the stable 2.5 V (*V*_DD_/2) bias generated internally by the AD5933′s transimpedance amplifier.

Following this amplification and biasing stage, the conditioned voltage output is routed through a series resistor, designated as RCURR2, before being fed directly into the pin of the AD5933. This specific series resistor acts as a critical voltage-to-current converter, ensuring the amplified signal is appropriately formatted as a current input for the AD5933′s internal transimpedance amplifier. To complete this analog front-end configuration, a feedback resistor (RESRFB) is externally connected in parallel between the and RFB pins of the AD5933. This works in tandem with the internal TIA to form a robust current-to-voltage gain stage, allowing users to safely utilize the full dynamic range of the internal ADC [13].

### 7.3 Signal to noise ratio evaluation

To evaluate the signal stability and the noise suppression capability of the system, a Signal-to-Noise Ratio (SNR) analysis was conducted. The evaluation compared four combinations of Voltage-Controlled Current Source (VCCS) and instrumentation amplifier (IA): LOP-INA118, LOP-AD620, HOW-INA118, and HOW-AD620. The system was configured using an adjacent excitation and measurement strategy on a 16-electrode phantom, applying a 1 Vpp, 50.68 kHz signal. The evaluation was conducted using a homogeneous medium without any target inclusions, yielding 208 impedance data per frame. A total of 50 consecutive frames (N) were acquired for each VCCS configuration.

For each channel k(-), the mean signal magnitude ${\overset{¯}{V}}_{k}\left(V\right)$ and the standard deviation $\sigma_{k}$ (V) over N(-) consecutive frames were calculated using Equation (5) and Equation (6), respectively, where $V_{k,i}\left(V\right)$ represents the measured boundary voltage of channel k(-), at frame i(-). Finally, the SNR in decibels (dB) was determined using Equation (7).

(5) $${\overset{¯}{V}}_{k}=\frac{1}{N}\sum_{i=1}^{N}V_{k,i}$$

(6) $$\sigma_{k}=\sqrt{\frac{1}{N-1}\sum_{i=1}^{N}{\left(V_{k,i}-{\overset{¯}{V}}_{k}\right)}^{2}}$$

(7) $$\mathrm{SNR}=20\times \log_{10}\left(\frac{\left|{\overset{¯}{V}}_{k}\right|}{\sigma_{k}}\right)$$

**Fig. 12:**
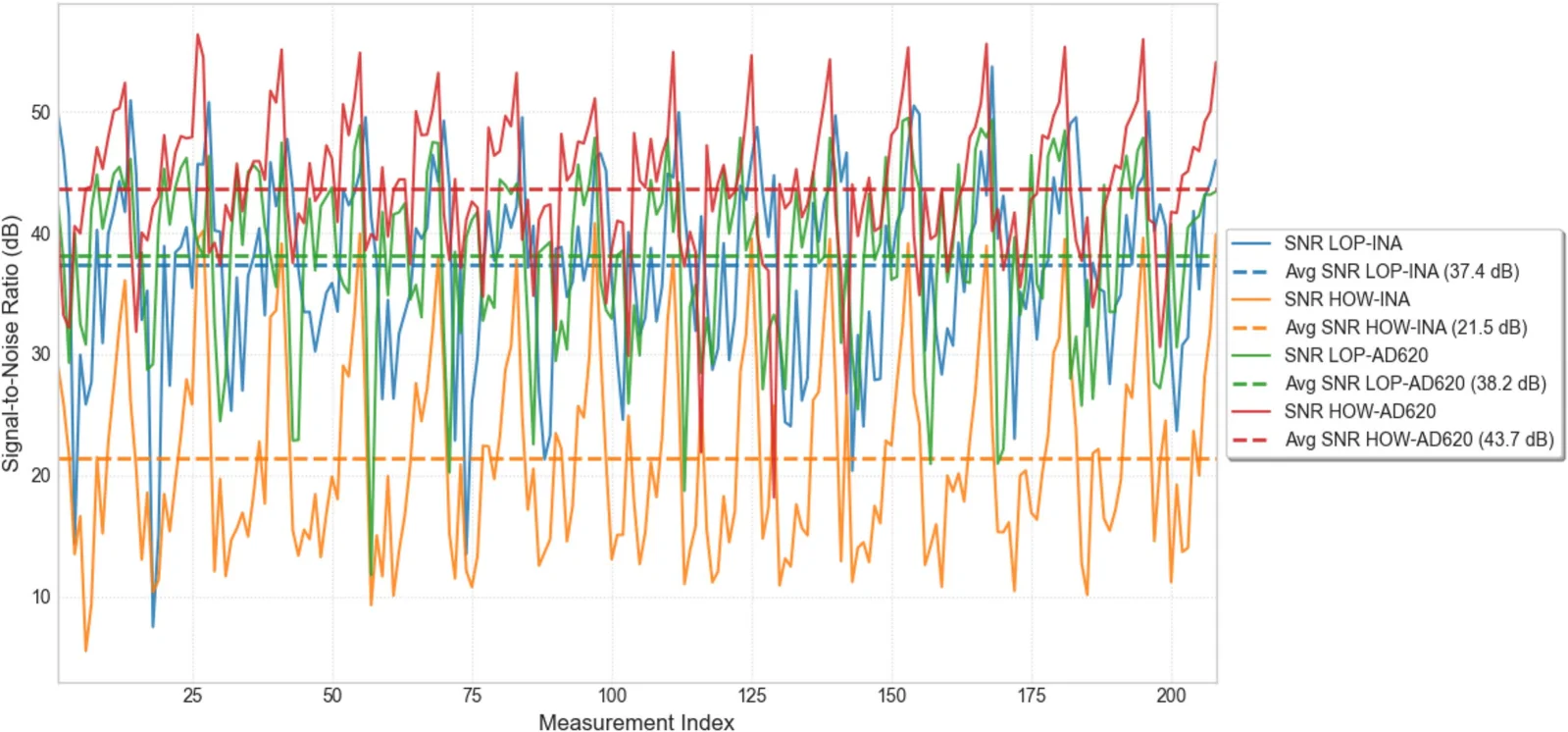
Signal-to-noise-ratio (SNR) for each VCCS and IA configurations.

A periodic ripple pattern was observed in the SNR curves across all configurations in the Fig. 12. This is a characteristic electrical behavior of the adjacent measurement multiplexer swithcing method. Channels located geometrically closer to the active current injection electrodes capture higher boundary voltages, resulting in high SNR peaks. Conversely, measurements taken at the opposite side of the phantom domain furthest from the injection site yield significantly weaker differential voltages. These far-field channels are more susceptible to background electronic noise, which corresponds to the periodic drops in the SNR curve.

Physically, the LOP topology is structurally simpler and does not depend on symmetrical resistor matching. By placing the phantom load directly within the operational amplifier's negative feedback loop, LOP guarantees a stable and consistent current injection even when using standard off-the-shelf components. Conversely, the Mirrored Howland current pump fundamentally relies on strict resistor ratio symmetry to achieve high output impedance. In a low-cost setup utilizing standard 1% tolerance resistors, any component mismatch prevents the HOW circuit from maintaining a stable output impedance [16]. This structural difference explains the distinct behavior of each configuration, while the LOP-based setups yield highly stable and uniform channel-wide SNR measurements (with a 37.4 dB for LOP-INA and 38.2 dB for LOP-AD620), the HOW-based setups exhibit extreme sensitivity to the paired instrumentation amplifier. Consequently, the HOW-AD620 configuration achieved the highest peak SNR of approximately 43.7 dB, whereas the HOW-INA118 configuration yielded the poorest noise performance of approximately 21.5 dB.

To evaluate these results, our system's SNR range spanning from 21.5 dB to 43.7 dB across all configurations is adequate for low-cost EIT applications and comparable to pioneer clinical platforms. For instance, the clinically validated Sheffield Mk 3.5 system operated on a baseline SNR of approximately 40 dB [24]. Furthermore, in recent open-source EIT hardware developments, Creegan et al. reported channel SNRs spanning 36–63 dB [5], and Brazey et al. noted that several active channels routinely dropped to 35 dB without degrading the final image quality [25]. While high-end academic platforms like the Canterbury CUBE system reach an average SNR of 85 dB [7], they require complex multi-board stacking and cost three times more (∼330 USD). Because our system utilizes time-difference imaging, static hardware offsets, parasitic capacitances, and common-mode noise are mathematically subtracted. This algorithmic tolerance ensures that our SNR span is robust enough to reconstruct high-contrast anomalies with minimal noise propagation.

### 7.4 System accuracy and repeatability validation

To evaluate the performance and stability of the proposed EIT system, a hardware validation test was conducted using six known resistors ranging from 100 Ω, 220 Ω, 328 Ω, 470 Ω, 506 Ω, 676 Ω, and 1000 Ω. Prior to the measurement, a calibration was performed to derive the system's gain factor. To assess the measurement repeatability, the microcontroller was programmed to record N = 50 consecutive impedance samples for each target resistor through the active multiplexer channels.

The acquired datasets were analyzed to determine both the precision and the absolute accuracy of the hardware. The mean measured impedance ${\overset{¯}{Z}}_{m}\left(\Omega \right)$ across the N(-) samples was calculated using Equation (8), as follows:

(8) $${\overset{¯}{Z}}_{m}=\frac{1}{N}\sum_{i=1}^{N}Z_{i}$$

where $Z_{i}$ is *i*-th measured impedance (Ω).

The measurement stability and noise floor of the system, represented by the standard deviation σ(Ω), were evaluated using Equation (9):

(9) $$\sigma =\sqrt{\frac{1}{N-1}\sum_{i=1}^{N}{\left(Z_{i}-{\overset{¯}{Z}}_{m}\right)}^{2}}$$

Furthermore, to quantify the hardware's measurement accuracy against the reference values, $Z_{k}\left(\Omega \right)$, the absolute error $E_{\mathrm{absolute}}$ (Ω), and the percentage of relative error $E_{\mathrm{relative}}\left(\%\right)$ were determined using Equation (10) and Equation (11), respectively:

(10) $$E_{\mathrm{absolute}}=\left|{\overset{¯}{Z}}_{m}-Z_{k}\right|$$

(11) $$E_{\mathrm{relative}}=\left(\frac{\left|{\overset{¯}{Z}}_{m}-Z_{k}\right|}{Z_{k}}\right)\times 100\%$$

The statistical analysis of the recorded samples reveals that the implemented hardware exhibits exceptional precision. As summarized in Table 5, the standard deviation for all tested resistive loads remains remarkably low across all configurations, ranging between 0.54 Ω and 6.33 Ω. This tight clustering of data points indicates a highly stable VCCS and minimal background noise within the signal conditioning circuit.

**Table 5:** Proposed System Accuracy Test Result.

**Configuration**	**Nominal Resistance,**$Z_{k}$**(Ω)**	**Mean Measured,**${\overset{¯}{Z}}_{m}$**(Ω)**	**Standard Deviation,**σ**(Ω)**	**Absolute Error,**$E_{absolute}$**(Ω)**	**Relative Error,** $E_{relative}$**(%)**
**HOW-AD620**	100	103.91	1.29	3.91	3.91
	220	224.13	1.28	4.13	1.88
	328	333.62	1.73	5.62	1.71
	470	475.61	1.66	5.61	1.19
	506	511.34	1.37	5.34	1.06
	676	680.05	1.36	4.05	0.6
	1000	983.86	2.07	16.14	1.61
**HOW-INA118**	100	109.77	4.97	9.77	9.77
	220	226.59	4.58	6.59	2.99
	328	332.49	6.33	4.49	1.37
	470	475.38	5.83	5.38	1.15
	506	567.66	0.64	61.66	12.19
	676	727.49	0.7	51.49	7.62
	1000	986.19	4.45	13.81	1.38
**LOP-AD620**	100	137.82	0.73	37.82	37.82
	220	284.62	0.77	64.62	29.37
	328	433.31	0.72	105.31	32.11
	470	574.41	0.78	104.41	22.21
	506	611.98	0.76	105.98	20.94
	676	690.13	0.86	14.13	2.09
	1000	987.71	0.9	12.29	1.23
**LOP-INA118**	100	122.88	0.63	22.88	22.88
	220	264.51	0.61	44.51	20.23
	328	384.85	0.59	56.85	17.33
	470	532.49	0.6	62.49	13.3
	506	567.47	0.6	61.47	12.15
	676	727.17	0.54	51.17	7.57
	1000	985.5	0.58	14.5	1.45

**Fig. 13:**
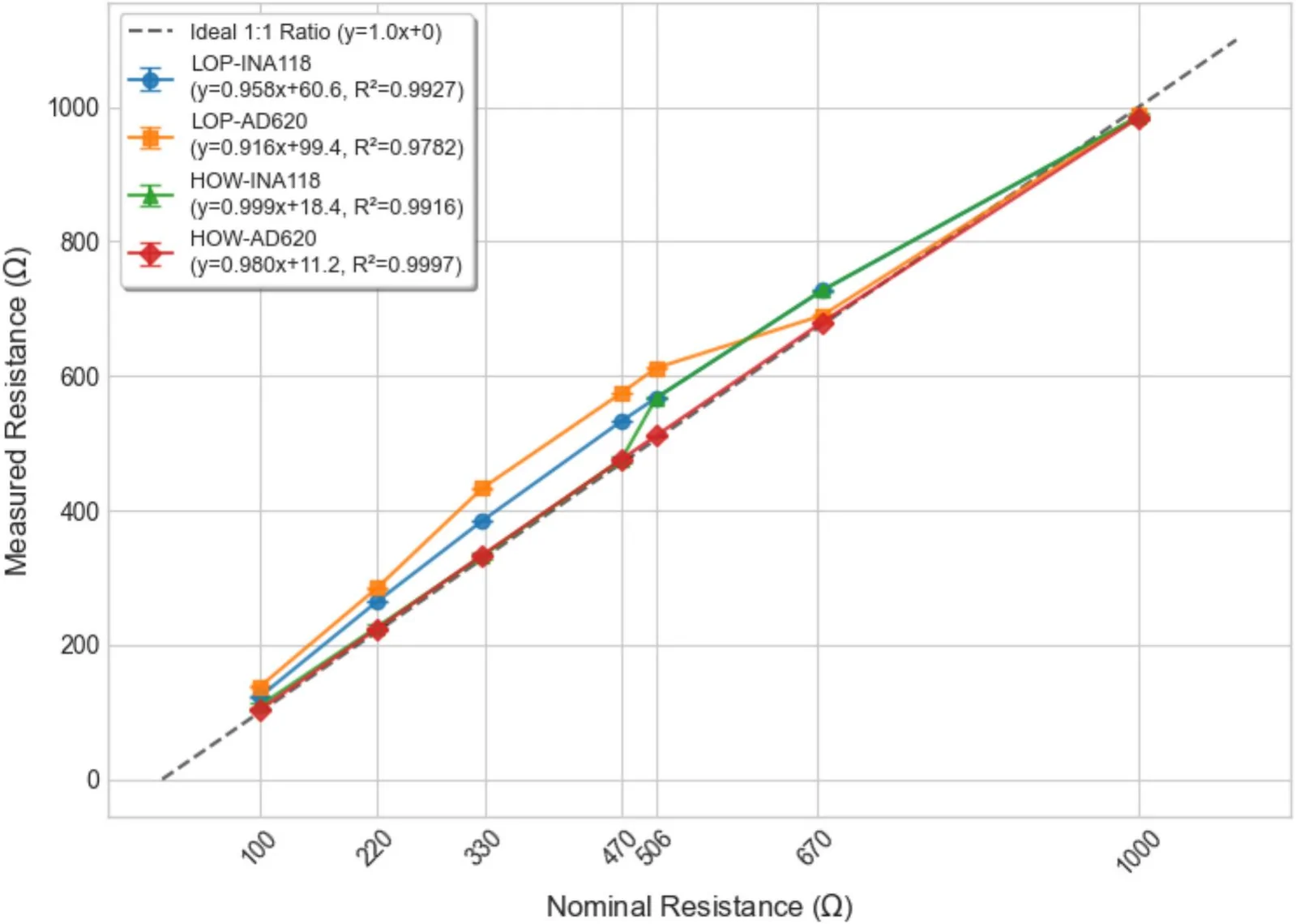
Measured vs. Nominal Resistance value.

Furthermore, the linearity plot in Fig. 13 demonstrates that the measured impedance closely follows the ideal 1:1 reference line across the entire evaluated spectrum. To quantitatively evaluate this correlation and investigate the presence of systematic bias, a linear regression analysis (y=mx+c) was conducted on the measurement data. The regression yielded high coefficients of determination ($R^{2}$) ranging from 0.9782 to 0.9997, with slopes (m) closely approaching unity, ranging from 0.916 to 0.999. This confirms that the system can reliably and proportionately detect changes in load impedance a crucial requirement for generating accurate time-difference EIT images. However, the regression analysis also extracted positive y-intercepts (c) ranging from 11.2 Ω to 99.4 Ω, which mathematically confirms the presence of a fixed additive error inherent within the analog front-end circuitry.

**Fig. 14:**
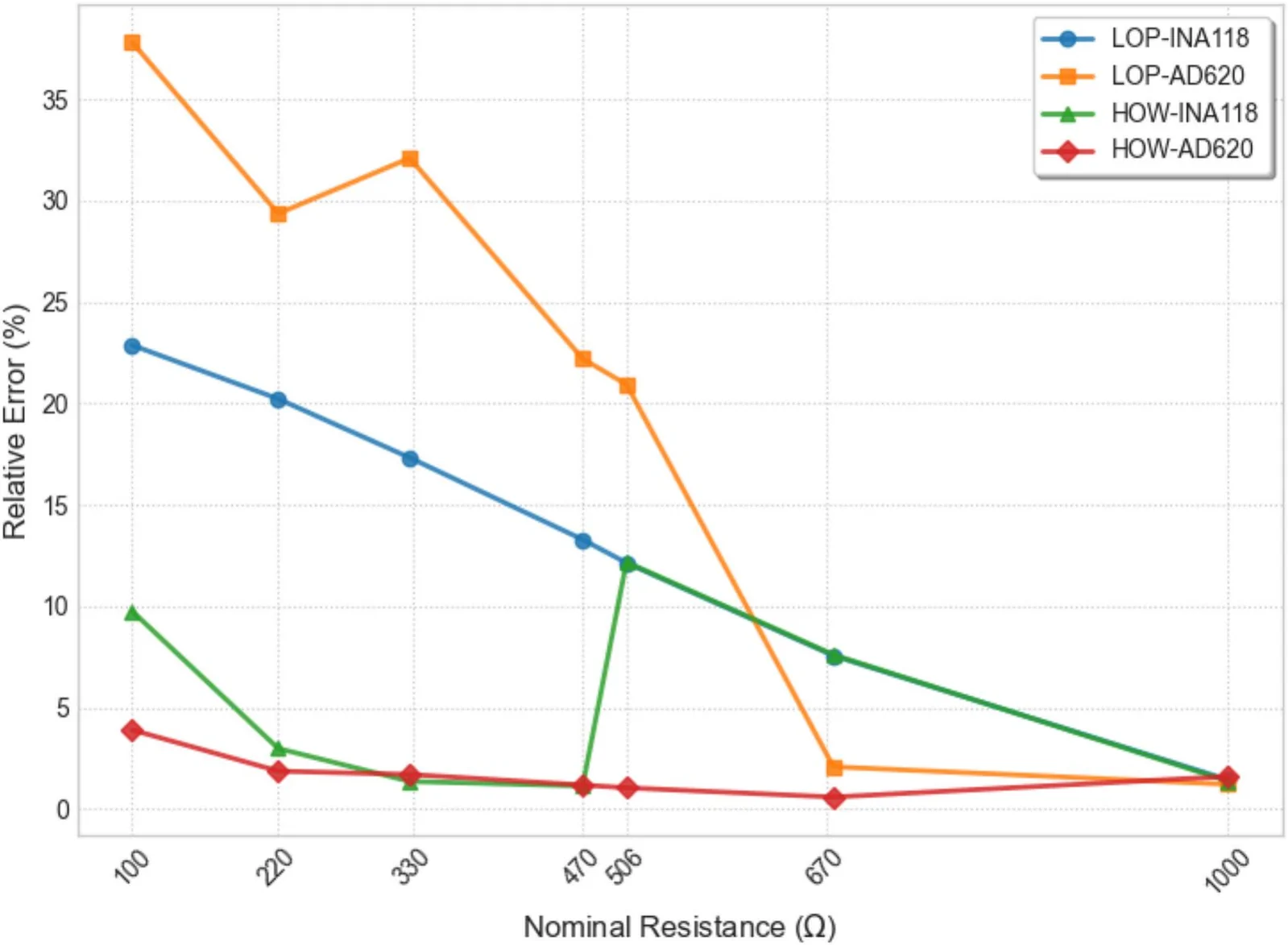
System Accuracy from experiments with various resistive loads.

Regarding absolute accuracy, this systematic bias manifests as a consistent positive static offset across measurements, yielding relative errors that span from 0.60% to 37.82% as depicted in Table 5 and Fig. 14. Importantly, for Electrical Impedance Tomography (EIT) applications that utilize time-difference image reconstruction algorithms, absolute impedance accuracy is secondary. Static hardware offsets and fixed additive errors are systematically cancelled out during the baseline subtraction phase of the reconstruction process. Therefore, high repeatability demonstrated by this system stands as the critical metric, guaranteeing that the hardware is fully capable of capturing differential voltage changes required for robust EIT data acquisition.

### 7.5 Image reconstruction

Table 6, Table 7, Table 8, Table 9 present the EIT image reconstruction results generated by the proposed system of the four different VCCS and instrumentation amplifier configurations. The experimental validation was conducted within a homogeneous saline water background medium at a concentration of 5 g/L. The conductivity of this medium was experimentally verified to be 966 mS/m at 25°C using a Lutron CD4303 conductivity meter. Within this conductive environment, non-conductive plastic tube phantoms with a diameter of 4 cm were the target anomalies. Back-Projection (BP) was chosen for its rapid reconstruction speed and low computational cost. Compared to iterative Jacobian-based (JAC) methods, which are computationally expensive and time-consuming, especially with high-density meshes. BP was fast and reliable image generation suitable for this hardware evaluation.

**Table 6:** Real condition, Reference Image generated by pyEIT, and Reconstructed Image with LOP-INA118 confguration.

**Table 7:** Real condition, Reference Image generated by pyEIT, and Reconstructed Image with HOW-INA118 configuration.

As detailed in the Table 6, Table 7, Table 8, Table 9, the proposed EIT system was evaluated under four spatial positions. The “Reconstructed Image” row displays the output of a single measurement frame. To optimize the signal-to-noise ratio (SNR), this frame is derived from a localized averaging technique, the AD5933 sequentially captures three impedance readings for each channel, which are then calculated and averaged by the onboard microcontroller before being transmitted to the personal computer (PC) [5]. This systematic acquisition yields a complete set of 208 impedance data measurements required to reconstruct a single 2D EIT image. Furthermore, the “Reference Image” row provides the ground truth simulated via the pyEIT forward model, mirroring the spatial positions of the phantoms under actual experimental conditions.

**Table 8:** Real condition, Reference Image generated by pyEIT, and Reconstructed Image with LOP-AD620 configuration.

**Table 9:** Real condition, Reference Image generated by pyEIT, and Reconstructed Image with HOW-AD620 configuration.

To quantitatively evaluate the quality of these reconstructed EIT images against the simulated ground truth, the Structural Similarity Index Measure (SSIM) was calculated. SSIM designed to evaluate the visual impact of shifts in image luminance, contrast, and structure, closely mimicking human visual perception. The SSIM(-) between the Reference image x and the Reconstructed image y is calculated using Equation (12).

(12) $$\mathrm{SSIM}\left(x,y\right)=\frac{\left(2\mu_{x}\mu_{y}+C_{1}\right)\left(2s_{\mathrm{xy}}+C_{2}\right)}{{(\mu }_{x}^{2}+\mu_{y}^{2}+C_{1})+\left(s_{x}^{2}+s_{y}^{2}+C_{1}\right)}$$

where $\mu_{x}$ and $\mu_{y}$ are the mean pixel intensities of images x(-) and y(-), $s_{x}^{2}\left(-\right)$ and $s_{y}^{2}\left(-\right)$ are the pixel intensity variance, $s_{\mathrm{xy}}\left(-\right)$ is the covariance, and $C_{1}\left(-\right)$ and $C_{2}\left(-\right)$ are stabilization constants.

In addition, the Root Mean Square Error (RMSE), RMSE(-) was calculated to quantitatively assess the absolute differences in pixel intensities between the reconstructed image and the ground truth image. While SSIM focuses on visual perception and spatial structure, RMSE provides a mathematical measure of the reconstruction accuracy by penalizing larger magnitude errors or artifacts. The RMSE is calculated using Equation (13):

(13) $$\mathrm{RMSE}=\sqrt{\frac{1}{N}\sum_{i=1}^{N}{\left(x_{i}-y_{i}\right)}^{2}}$$

where $x_{i}\left(-\right)$ is the pixel intensity value of the reference ground-truth image at pixel index i(-), $y_{i}\left(-\right)$ is the pixel intensity value of the reconstructed image and N(-) is the total number of pixels in the image mesh.

The experimental reconstruction results demonstrate that the proposed EIT system can detect and localize the spatial positions of both highly conductive (stainless steel) and non-conductive (plastic) target anomalies under all evaluated configurations. However, as observed in the reconstructed images, the detected inclusions do not exhibit perfect circular shapes as shown in the simulated reference images, instead displaying spatial deformation and boundary smearing. This deformation is a characteristic physical behavior of EIT systems utilizing the adjacent current pattern combined with the linear Back-Projection (BP) algorithm, which is inherently subject to spatial resolution limits, particularly as sensitivity degrades toward the center of the imaging domain. To provide a rigorous quantitative evaluation, the reference images were simulated and reconstructed using the pyEIT 2D Finite Element Method (FEM) forward solver to serve as the ground truth for Structural Similarity Index Measure (SSIM) and Root Mean Square Error (RMSE) calculations.

**Fig. 15:**
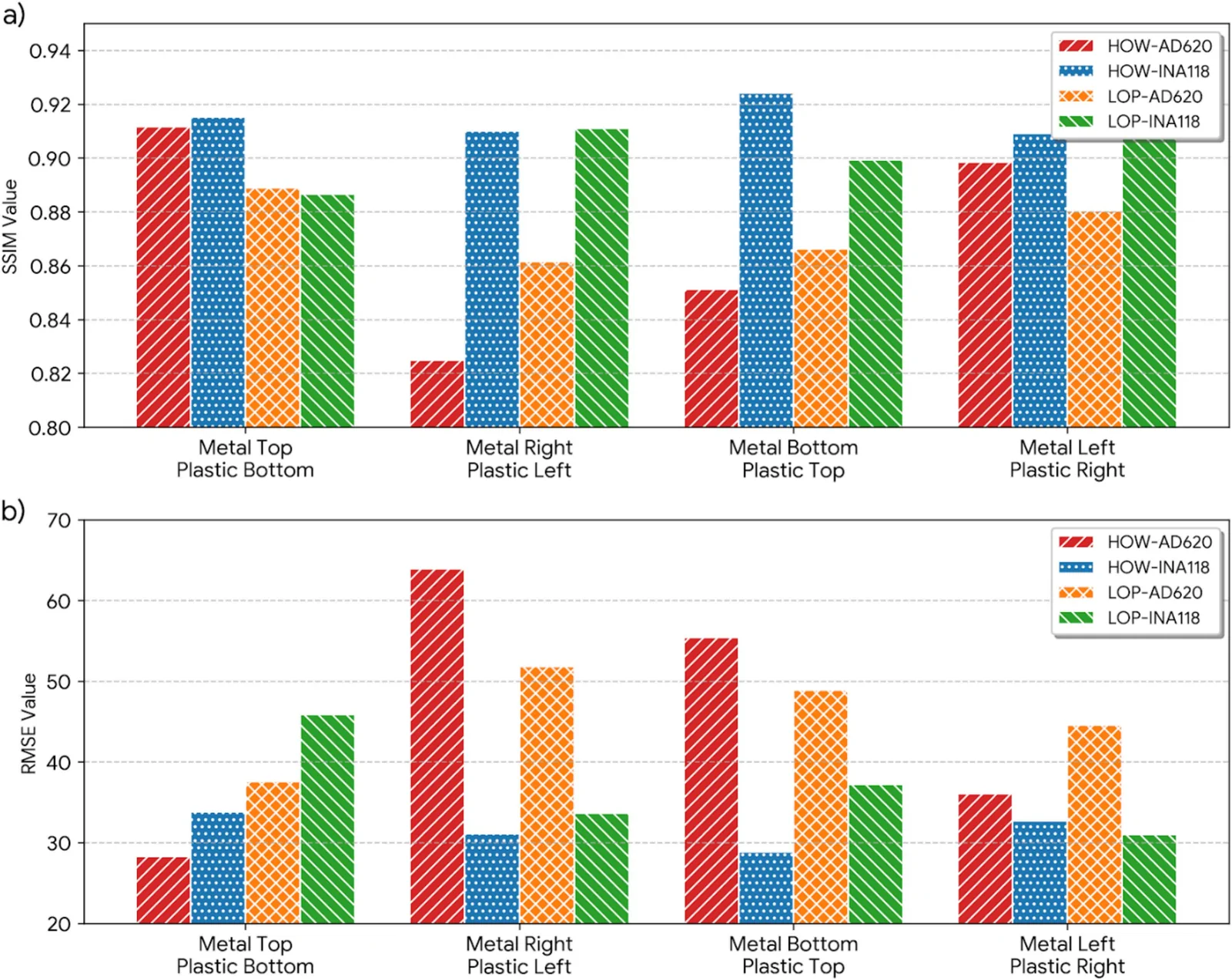
Comparison of the image reconstruction quality across all evaluated VCCS and IA combinations for four distinct target positions: (a) Structural Similarity Index Measure (SSIM) values, and (b) Root Mean Square Error (RMSE) values.

Fig. 15 illustrates the comparison of the SSIM and RMSE values across all four modular hardware configurations and all varied phantom positions. The quantitative metrics highlight that the HOW-INA118 configuration achieved the highest overall reconstruction fidelity, yielding a peak SSIM of 0.9242 and a low RMSE of 28.85 when the stainless steel target was positioned at the bottom and the plastic target at the top. Additionally, the HOW-AD620 configuration demonstrated pixel intensity accuracy, achieving the lowest overall RMSE of 28.29 with a SSIM of 0.9117 under the same target placement. In comparison, the LOP-AD620 configuration exhibited more moderate results, with SSIM values ranging from 0.8615 to 0.8890 and RMSE values spanning 37.55 to 51.81. These quantitative evaluations confirm that despite the circular distortions, the developed modular system provides a robust and reliable platform for mapping extreme conductivity contrasts across diverse spatial orientations.

The acronyms used in this manuscript are compiled in Table 10. In addition, Table 11 defines the mathematical symbols and notations.

**Table 10:** Nomenclature.

**Acronym**	**Unit**	**Description**
LOP	-	Load-in-the-loop Voltage Controlled Current Source
HOW	-	Mirrored Howland Voltage Controlled Current Source
INA118	-	INA118 Instrumentation Amplifier
AD620	-	AD620 Instrumentation Amplifier
SNR	dB	Signal-to-noise-ratio
SSIM	-	Structural Similarity Index Measure ranging from −1 to 1.

**Table 11:** Mathematical Symbols.

**Variable**	**Unit**	**Description**
$V_{\mathrm{in}}$	V	Input voltage signal to VCCS
$I_{\mathrm{out}}$	A	Excitation current generated by the VCCS
G	-	Voltage gain of instrumentation amplifier
$R_{g}$	Ω	External gain-setting resistor used to configure G
N	-	Total number of recorded data frames or samples per measurement session.
k	-	Index of measured impedance data, (k = 1, 2, …, 208).
i	-	Index of the individual sample or data frame, (i = 1, 2, …, N).
${\overset{¯}{V}}_{k}$	V	Mean of the measured voltage signal at channel k over N
$V_{k,i}$	V	Measured boundary voltage at channel k for frame i
σ	Ω	Standard deviation of the acquired sample
$\sigma_{k}$	V	Standard deviation of the acquired frame
${\overset{¯}{Z}}_{m}$	Ω	Mean of the measured impedance over N samples.
$Z_{i}$	Ω	Measured impedance magnitude at sample index i.
$Z_{k}$	Ω	Nominal of the resistor used for validation.
$E_{\mathrm{absolute}}$	Ω	Absolute measurement error
$E_{\mathrm{relative}}$	%	Relative error
$\mu_{x}$, $\mu_{y}$	-	Average pixel intensity of the evaluated image.
$s_{x}^{2}$, $s_{y}^{2}$	-	Sample variance representing the global contrast of an image.
$s_{\mathrm{xy}}$	-	Sample covariance indicating structural correlation between two images.
$C_{1}$, $C_{2}$	-	Small constants used to stabilize the SSIM mathematical division.
$x_{i}$	-	Pixel intensity value of the reference ground truth image
$y_{i}$	-	Pixel intensity value in the reconstructed image

### 7.6 Summary

The EIT system developed in this research successfully provides a portable, low-cost, and highly modular 16-electrode imaging modality utilizing the AD5933 impedance converter and an STM32 microcontroller. The system incorporates robust signal conditioning interface circuits specifically designed to form a four-terminal sensing architecture. To achieve this, the front-end uniquely incorporates interchangeable VCCS utilizing either a Load-in-the-Loop current source (LOP) or a Mirrored Howland current source (HOW) topology for current excitation, paired with interchangeable instrumentation amplifiers an INA118P IC or an AD620 module for boundary voltage amplification. To prevent signal clipping during data acquisition, a DC biasing mechanism is actively applied. Multiplexers were used to route the excitation and boundary voltage. Furthermore, custom pyEIT- and Tkinter-based software was created for data acquisition and reconstruct 2D EIT images.

Experimental characterizations were conducted across all four modular hardware configurations (LOP-INA118, LOP-AD620, HOW-INA118, and HOW-AD620). SNR evaluations under homogeneous saline conditions (5 g/L, 966 mS/m at 25°C) demonstrated a maximum peak SNR of 43.7 dB under the HOW-AD620 configuration. System accuracy and repeatability tests using standard resistors (100–1000 Ω) revealed exceptional hardware precision, with measurement standard deviations ranging from 0.54 Ω to 6.33 Ω across all configurations. Although a systematic positive offset led to relative errors of 0.60% to 37.82%, this fixed additive error was mathematically cancelled during the baseline subtraction phase of the time-difference reconstruction process.

Finally, 2D image reconstruction capabilities were validated using both non-conductive plastic and highly conductive stainless steel target anomalies under various spatial position. Quantitative image quality analysis across all four configurations yielded reconstruction fidelity, achieving a maximum Structural Similarity Index Measure (SSIM) of up to 0.9242 with HOW-INA118 configuration and a minimum Root Mean Square Error (RMSE) of 28.29 (HOW-AD620 configuration). Additionally, the firmware and data pipeline complete 208 impedance data acquisition in 2 s, making it a suitable for EIT research and education.

### 7.7 Future work

While the proposed modular EIT system successfully achieves its design goals for educational and preliminary research purposes, several ideas for future hardware and software developments have been identified to further elevate its performance:

1. Hardware Upgrade and Real-Time Data Flow: To overcome the hardware bottlenecks of the AD5933, such as unipolar ADC constraints, limited DDS frequency sweep, and slower single-frequency sweeping, future iterations will explore upgrading to next-generation impedance converter chips with wider dynamic ranges and multi-frequency measurement capabilities. Furthermore, optimization of the microcontroller firmware and UART data transfer protocol will be executed to compress the acquisition time below 1 s, enabling true real-time EIT monitoring suitable for dynamic physiological applications such as respiration monitoring.
2. Signal-to-Noise Ratio (SNR) Optimization: Future hardware improvements will focus on elevating the SNR beyond the current maximum of 43.7 dB. This can be achieved by integrating active-shielded coaxial cables to suppress parasitic cable capacitances and implementing advanced digital filtering on the STM32 firmware.
3. Advanced Image Reconstruction via Generative Deep Learning: To overcome the inherent physical spatial resolution limits of EIT (especially near the center of the imaging domain), future work will investigate the integration of machine learning-based reconstruction frameworks. Specifically, generative models such as Conditional Variational Autoencoders (CVAEs) and Diffusion Models [26] will be implemented in the software pipeline. These models will be trained on simulated pyEIT datasets to enhance target boundary definition, improve accuracy, and reconstruct complex anomaly shapes with higher spatial resolution.

### CRediT authorship contribution statement

**Jerry Febrico:** Writing – original draft, Visualization, Validation, Software, Methodology, Investigation, Data curation, Conceptualization. **Basari:** Writing – review & editing, Supervision, Funding acquisition.

## Declaration of competing interest

The authors declare that they have no known competing financial interests or personal relationships that could have appeared to influence the work reported in this paper.
